# Proteomic analysis of excretory-secretory products from young adults of *Angiostrongylus cantonensis*

**DOI:** 10.1590/0074-02760180556

**Published:** 2019-06-19

**Authors:** Kuang-Yao Chen, Pei-Jhen Lu, Chien-Ju Cheng, Kai-Yuan Jhan, Shih-Chien Yeh, Lian-Chen Wang

**Affiliations:** 1 China Medical University, School of Medicine, Department of Parasitology, Taichung, Taiwan; 2 Chang Gung University, College of Medicine, Department of Parasitology, Taoyuan, Taiwan; 3 Chang Gung University, College of Medicine, Graduate Institute of Biomedical Sciences, Taoyuan, Taiwan; 4 Molecular Infectious Disease Research Centre, Chang Gung Memorial Hospital, Taoyuan, Taiwan

**Keywords:** Angiostrongylus cantonensis, young adult worms, excretory/secretory products, proteomic, immunoreactive proteins

## Abstract

**BACKGROUND:**

Angiostrongyliasis is caused by the nematode *Angiostrongylus cantonensis* and can lead to eosinophilic meningitis and meningoencephalitis in humans. The young adult worms play central pathogenic roles in the central nervous system (CNS); however, the underlying mechanism is unclear. Excretory-secretory products (ESPs) are good investigation targets for studying the relationship between a host and its parasite.

**OBJECTIVES:**

We aimed to profile, identify, and characterise the proteins in the ESPs of *A. cantonensis* young adults.

**METHODS:**

The ESPs of young adult worms were collected from culture medium after incubation ranging from 24 to 96 h. Proteomic and bioinformatics analyses were performed to characterise the ESPs.

**FINDINGS:**

A total of 51 spots were identified, and the highly expressed proteins included two protein disulphide isomerases, one calreticulin, and three uncharacterised proteins. Subsequently, approximately 254 proteins were identified in the ESPs of *A. cantonensis* young adults via liquid chromatography-mass spectrometry (LC-MS/MS) analysis, and these were further classified according to their characteristics and biological functions. Finally, we identified the immunoreactive proteins from a reference map of ESPs from *A. cantonensis* young adults. Approximately eight proteins were identified, including a protein disulphide isomerase, a putative aspartic protease, annexin, and five uncharacterised proteins. The study established and identified protein reference maps for the ESPs of *A. cantonensis* young adults.

**MAIN CONCLUSIONS:**

The identified proteins may be potential targets for the development of diagnostic or therapeutic agents for human angiostrongyliasis.

*Angiostrongylus cantonensis* is the rat lungworm, and a zoonotic parasitic nematode that causes eosinophilic meningitis and eosinophilic meningoencephalitis in humans.^1^ ^,^ ^2^ This nematode was found in the hearts and pulmonary arteries of rats (*R. rattus* and *R. norvegicus*) in Guangzhou (Canton), China by Dr. HT Chen in 1935,^3^ ^,^ ^4^ and the first human case was discovered in Taiwan in 1944.^5^

The complex life cycle of *A. cantonensis* requires a definitive host (rats) and an intermediate host (molluscan).^6^ ^,^ ^7^ ^,^ ^8^ The adult worms live and mate in the right ventricle and pulmonary arteries of rats. The eggs are produced from female worms and hatch to the first-stage larva (L1) in the lung blood capillaries. The larva then penetrates the alveolar capillaries and migrates to the throat. After entering the gastrointestinal tract, these larvae are released via rat faeces. The first-stage larvae in faeces may infect the intermediate host via skin penetration or through ingestion. After the definitive host feeds on the intermediate host or paratenic host containing the infective third-stage larvae (L3), the larvae penetrate the intestinal wall into the blood circulation to reach the central nervous system (CNS) and develop into young adult worms. At this stage, the larvae can induce mild or severe immune responses, mechanical injuries, and mortality outcomes in hosts.^9^

Recently, proteomic approaches that substantially improve the efficiency of protein analysis, even for low-abundance target proteins, have been developed. Proteomic analysis is used to detect changes in protein expression. Two-dimensional gel electrophoresis (2-DE) coupled with matrix-assisted laser desorption ionisation time-of-flight (MALDI-TOF) was previously employed to elucidate protein patterns and for their identification.^10^ This technique has been highly recommended for studies dealing with parasites, host responses, and host-parasite interactions. In *Plasmodium falciparum* infections, proteomic analysis has been employed to verify expression changes at the different developmental stages, such as asexual blood stages and gametocytes.^11^ ^,^ ^12^ ^,^ ^13^

Excretory-secretory products (ESPs) are valuable targets for investigation of host-parasite relationships. These products contain a wide range of molecules, including proteins, glycans, lipids, and nucleic acids, that aid in the penetration of host defensive barriers and avoidance of host immune attack in nematodes, trematodes, and cestodes.^14^ In our previous study, we demonstrated that apoptosis, oxidative stress, and cytokine secretion were induced in mouse astrocytes treated with the ESPs of *A. cantonensis* young adults.^15^ ^,^ ^16^ Recent findings have revealed that ESPs could be secreted from *A. cantonensis* adult worms and could stimulate host immune responses. Some of these secretory proteins include heat shock protein 70, aspartyl protease inhibitor, cathepsin B-like cysteine proteinase, and haemoglobinase-type cysteine proteinase,^17^ and these proteins have been implicated in host infections. In our previous study, we used 2-DE and MALDI-TOF to confirm somatic protein expression, as well as for protein identification in the third-stage larvae and the young adults of *A. cantonensis*. We showed that approximately 15 protein spots were stress-related proteins, and identified heat shock protein 60 as the most highly expressed heat stress protein in the young adults.^18^ In the present study, we obtained the ESPs from the young adults and determined the expression profiles of the different proteins in the secretion. We show, via bioinformatics analysis, that the highly expressed proteins have potential functions in cell survival, development, and host immune response resistance. We present predicted targets for further investigation of the mechanisms of nematode infection and stress adaptation.

## MATERIALS AND METHODS

*Ethics* - All animal protocols in this study were approved by the Chang Gung University Institutional Animal Care and Use Committee (CGU15-033; CGU15-067). Rats and mice were housed in plastic cages and provided with food and water ad libitum. The experimental animals were sacrificed by anaesthesia with 3% (v/v) isoflurane (Panion & BF Biotech Inc., Taipei, Taiwan).

*Animals and parasite infection* - In this study, the *A. cantonensis* strain used had been maintained in our laboratory since 1980 and had been cycling through Sprague-Dawley (SD) rats and *Biomphalaria glabrata* snails.^15^ SD rats were purchased from the BioLASCO, Taipei. These rats were maintained in the Laboratory Animal Centre according to guidelines approved by the Chang Gung University Institutional Animal Care and Use Committee (CGU15-033). To isolate L3, the infected *B. glabrata* snails were killed on day 21, and the tissues were homogenised with an organisation homogeniser (Cole-Parmer Instrument Co., USA) and then digested with artificial gastric juice (0.6% (w/v) pepsin-HCl, pH 2-3) at 37ºC for 45 min.^15^ The male rats (eight weeks old; weight, 250 g) were infected with 50 L3 by oral inoculation. The rats were sacrificed, and the young adults were collected from the brain tissue three weeks post-infection.

*ESP preparation* - After infecting 100 L3 to each rat, brain tissues were obtained after anesthetising with 3% (v/v) isoflurane on day 21 post infection.^19^ The living young adults were collected from the brains of hosts, examined, and removed of tissue debris carefully under a dissecting microscope. Worms were washed three times with saline, phosphate-buffered saline (PBS), distilled water, and RPMI containing a high concentration of antibiotic (2 × Antibiotic-Antimycotic Solution; Sigma-Aldrich, USA). After incubating in RPMI without foetal bovine serum (FBS) for 96 h (37ºC; 5% CO_2_), the culture medium was obtained and concentrated using the Amicon Ultra-15 10K centrifugal filter devices (Merck Millipore, Germany). The protein concentration of ESP-containing medium was determined using the Bio-Rad Protein Assay Kit (Bio-Rad, USA).

*2-DE* - Approximately 300 µg of the ESPs was diluted to a final volume of 300 µL in rehydration buffer containing a trace amount of bromophenol blue [8 M urea, 2% (w/v) CHAPS] and then applied to a 13-cm immobilised pH gradient (IPG) gel strip (GE Healthcare, UK), with a linear separation range of pH 3-10 for the first dimensional electrophoresis.^18^ Rehydration and isoelectric focusing were performed in the Ettan IPGphor II (GE Healthcare, UK) using the following settings: 30 V for 12 h, 50 V for 0.5 h, 100 V for 0.5 h, 250 V for 0.5 h, 500 V for 0.5 h, 1,000 V for 0.5 h, 4,000 V for 0.5 h; and gradient to 8,000 V for 45,000 Vh. The IPG strip was incubated in an equilibration buffer [50 mM Tris-HCl (pH 8.8), 6 M urea, 30% glycerol, 2% sodium dodecyl sulphate (SDS) and a trace amount of bromophenol blue] containing 1% (w/v) dithiothreitol for 15 min and then in an equilibration buffer containing 2.5% (w/v) iodoacetamide for 15 min. The IPG strips were separated by 15% sodium dodecyl sulfate polyacrylamide gel electrophoresis (SDS-PAGE) and injected with 0.5% (w/v) agarose solution.

This experiment was repeated at least three times.

*2-DE gel visualisation and image analysis* - After protein separation by 2-DE, the proteins were visualised by silver staining for image analysis according to the procedures described by Chen et al.^18^ The protein spots in the silver-stained 2-DE gel (pI 3-10) were detected and analysed with Phoretix™ 2D analysis software (Phoretix, UK). The relative expression or difference of each protein spot was verified by determining the percentage of the total spot intensity. The percentage volume of each protein was presented as the mean ± standard deviation (SD) for at least triplicate 2-DE gels. The statistical significance was confirmed using two-tailed Student’s *t*-test for unpaired samples.

*In-sol digestion* - The concentrated ESPs (25 μg) were first diluted in 50 mM ammonium bicarbonate (ABC) and then reduced with 10 mM dithiothreitol (DTT, Merck, Germany) at 56ºC for 45 min and 40 mM iodoacetamide (IAA, Sigma, USA) at 25ºC for 30 min. The samples were digested with sequencing-grade modified porcine trypsin (Promega, USA) at 37ºC for 16 h. The peptides were then desalted and dried by vacuum centrifugation and stored at -80ºC until use.

*Liquid chromatography-mass spectrometry (LC-MS/MS) analysis* - The mixtures of peptide were reconstituted in HPLC buffer A (0.1% formic acid), and a reverse-phase column (Zorbax 300SB-C18, 0.3 × 5 mm; Agilent Technologies, USA) was used. The peptides were separated on a homemade column (HydroRP 2.5 μm, 75 μm I.D. × 20 cm with a 15 μm tip) using a multistep gradient of HPLC buffer B (99.9% acetonitrile/0.1% formic acid) for 70 min. The LC equipment was coupled with a 2D linear ion trap mass spectrometer (Orbitrap ELITE; Thermo Fisher, USA) operated using Xcalibur 2.2 software (Thermo Fisher, USA). The full-scan MS was performed in the Orbitrap over a range of 400 to 2,000 Da and a resolution of 60,000 at m/z 400. Internal calibration was performed using the ion signal of [Si(CH3)2O]6H+ at m/z 536.165365 as lock mass. The 20 data-dependent MS/MS scan events were followed by one MS scan for the 20 most abundant precursor ions in the preview MS scan. The m/z values selected for MS/MS were dynamically excluded for 60 s, with a relative mass window of 15 ppm. The electrospray voltage was set to 2.0 kV, and the temperature of the capillary was set to 200ºC. MS and MS/MS automatic gain controls were set to 1,000 ms (full scan) and 200 ms (MS/MS) or 3 × 10^6^ ions (full scan) and 3 × 10^3^ ions (MS/MS) for maximum accumulated time or ions, respectively.

*Protein identification and functional analysis* - The analysis was conducted using Proteome Discoverer software (version 1.4, Thermo Fisher Scientific). The MS/MS spectra were searched with *A. cantonensis* as the reference in the UniProt database (14,858 sequences) using the Mascot search engine (Matrix Science, London, UK; version 2.5). For peptide identification, 10 ppm mass tolerance was permitted for intact peptide masses and 0.5 Da for CID fragment ions with allowance for one missed cleavage made from the trypsin digestion: oxidised methionine, and acetyl (protein N-terminal) as variable modifications and carbamidomethyl (cysteine) as the fixed modification. Peptide-spectrum matches (PSMs) were then filtered based on high confidence and Mascot search engine rank 1 of peptide identification to ensure an overall false discovery rate below 0.01. Proteins with single peptide hits were removed.

*Western blotting analysis* - The male BALB/c mice (eight weeks old; 22-27 g) were infected with 50 L3 by oral inoculation. The blood specimens were obtained by cardiac puncture three weeks post-infection. The sera were collected by centrifugation at 1,500 × *g* and 30 min. The expression levels of immunoreactive proteins were determined using a 12.5% 2-DE gel. Semidry transfer equipment was used to transfer the proteins in the gels to a nitrocellulose membrane. The membrane was blocked with BSA buffer and then incubated with 1:100 dilution of the mouse antiserum at 4ºC overnight. The membranes were incubated with rabbit anti-mouse IgG peroxidase antibody (Sigma-Aldrich, USA) for 1 h at room temperature. The results were then detected using ECL reagents.^20^

## RESULTS

**Fig. 1:**
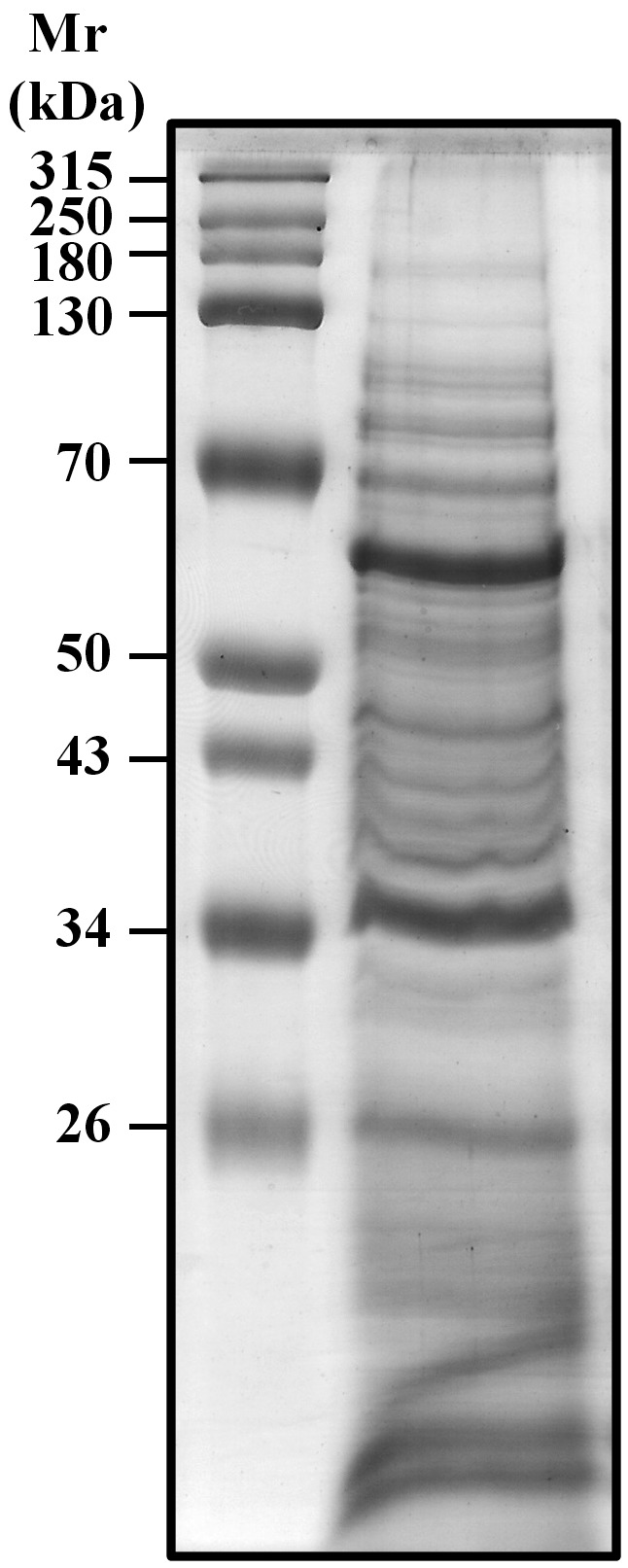
sodium dodecyl sulfate polyacrylamide gel electrophoresis (SDS-PAGE) of the excretory-secretory products (ESPs) from *Angiostrongylus cantonensis* young adults. Proteins were visualised by Coomassie blue staining.

**Fig. 2:**
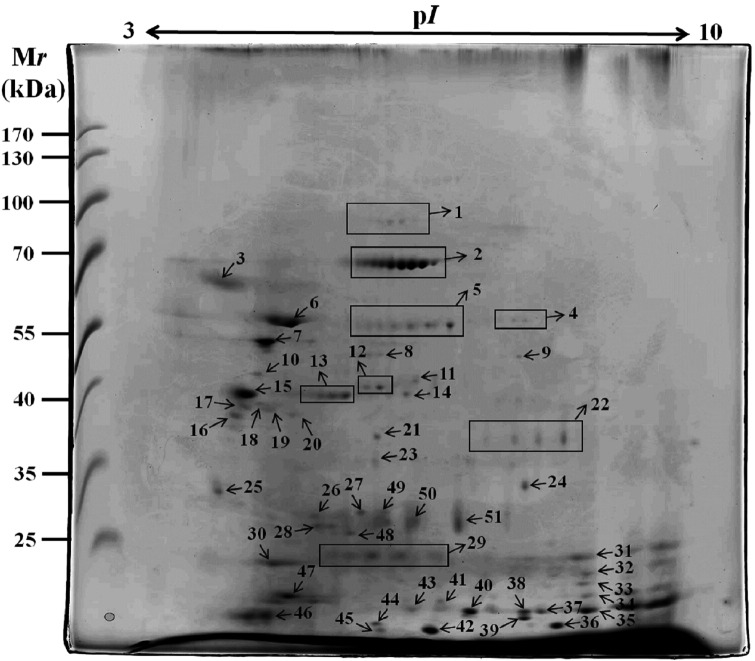
two-dimensional gel electrophoresis (2-DE) reference map of the excretory-secretory products (ESPs) of *Angiostrongylus cantonensis* young adults. The proteins were separated in the first dimension in the pH range 3-10 and in the second dimension on a 15% polyacrylamide gel. Proteins were visualised by silver staining.

*Proteome profile of the ESPs of A. cantonensis by 2-DE* - The ESPs of young adult worms were collected and concentrated from the no-serum RPMI culture medium at 37ºC under 5% CO_2_ and incubated for 24 to 96 h. The total proteins were separated by SDS-PAGE and visualised by Coomassie blue staining (Fig. 1). We initially established the global view of the protein expression profile of the ESPs of *A. cantonensis* young adults by 2-DE using an IPG strip of pH 3-10 (Fig. 2). Approximately 60 protein spots were detected in the reference map by silver staining, and most of the proteins were located between pH 4 and pH 8 with molecular weights between 0 and 100 kDa.

**Fig. 3:**
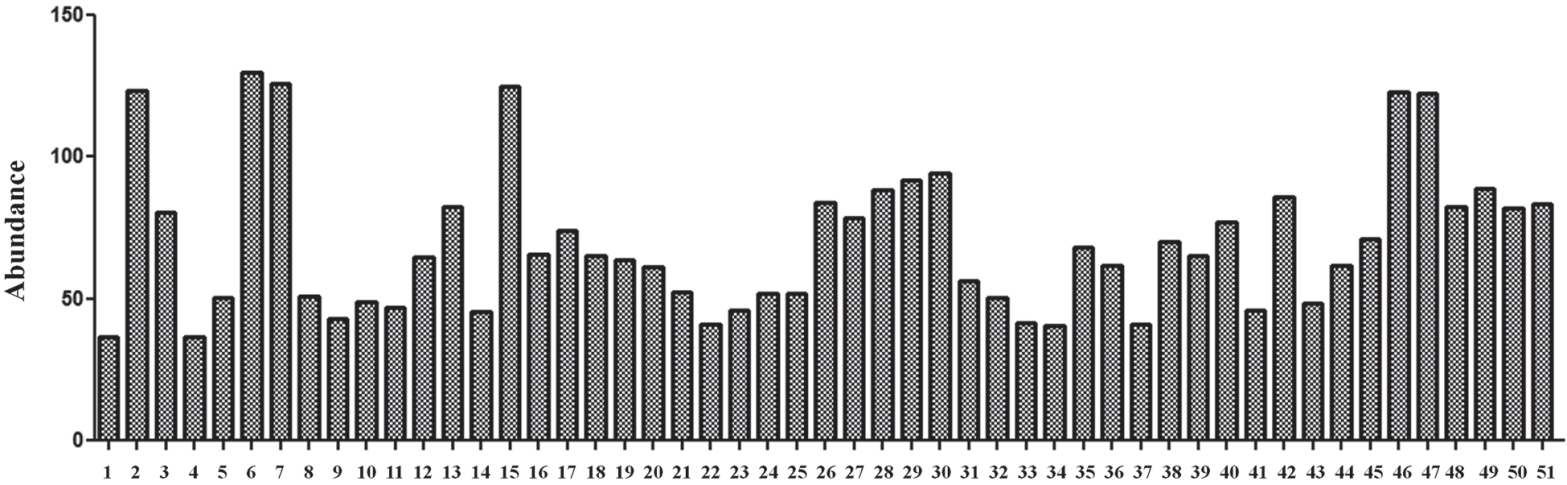
expression levels of 51 protein spots in the two-dimensional gel electrophoresis (2-DE) reference map. Each bar indicates the relative abundance quantified using ImageJ analysis software.

*Identity of total proteins in the reference map* - To identify the protein spots of *A. cantonensis* ESPs, the excised gel spots were destained and digested in-gel (In-sol digestion). A total of 51 protein spots were successfully identified by MALDI-TOF MS analysis [Supplementary data (Table I)], and the expression levels of the protein spots were also determined (Fig. 3). The most abundant proteins in the ESP map were protein disulphide isomerase (Spots 2 and 6), calreticulin (Spot 7), and uncharacterised proteins (Spots 15, 46, and 47). Subsequently, we found that the highest number of protein spots identified was that of protein disulphide isomerase (*n* = 8), peptidyl-prolyl cis-trans isomerase (*n* = 3), putative aspartic protease (*n* = 3), galectin (*n* = 2), and calreticulin (*n* = 2). The protein accession number (Accession), description (UniProt) and (NCBI protein database), peak area of the identified peptide (Area), protein score (Score), number of amino acid of identified protein (#AAs), and molecular weight [MW (kDa)] are shown in the supplementary results [Supplementary data (Table I)]. Moreover, to obtain the global protein identity of *A. cantonensis* ESPs, we used LC-MS/MS to establish the total ESP proteome. Approximately 254 proteins were identified in this manner [Supplementary data (Table II)].

**Fig. 4:**
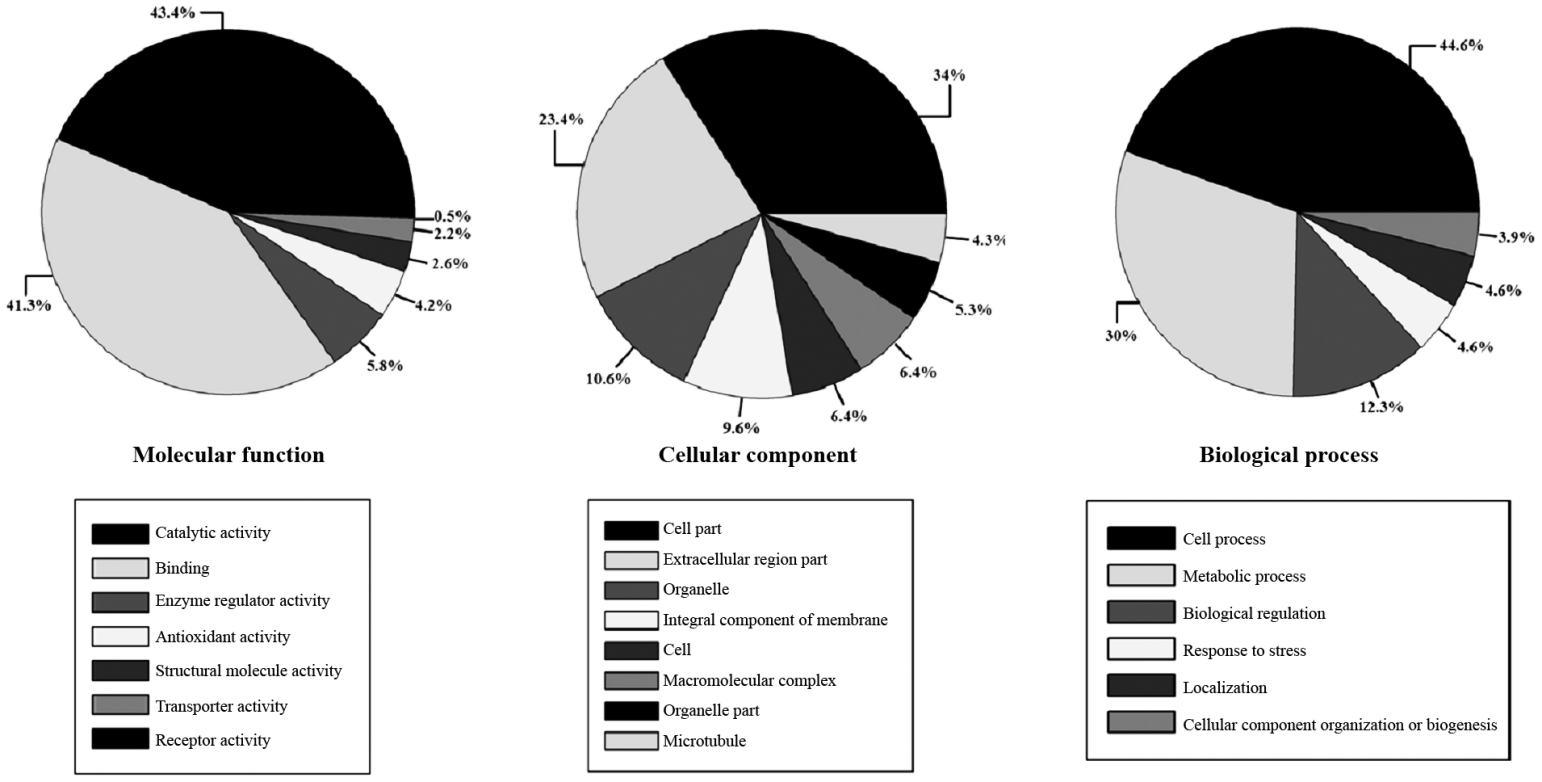
functional annotations of the proteins from the excretory-secretory products (ESPs) from *Angiostrongylus cantonensis* young adults based on Gene Ontology categories. The pie charts show the general categories: biological process, cellular component, and molecular function.

*Biological functions of the identified proteins* - The putative functional annotations of the identified protein spots by LC-MS/MS were explored and classified using the Gene Ontology (GO) database (http://www.geneontology.org/). Approximately 281 functions in 254 proteins were obtained, under molecular function (*n* = 141), cellular components (*n* = 67), and biological processes (*n* = 73) (Fig. 4). A total of eight proteins had antioxidant activity [GO:0016209], including glutathione peroxidase activity (*n* = 4) [GO:0004602], superoxide dismutase activity (*n* = 2) [GO:0004784], and peroxiredoxin activity (*n* = 2) [GO:0051920]. Moreover, we also detected various stress (*n* = 6) [GO:0006950] and oxidative stress (*n* = 4) [GO:0006979] proteins.

**Fig. 5:**
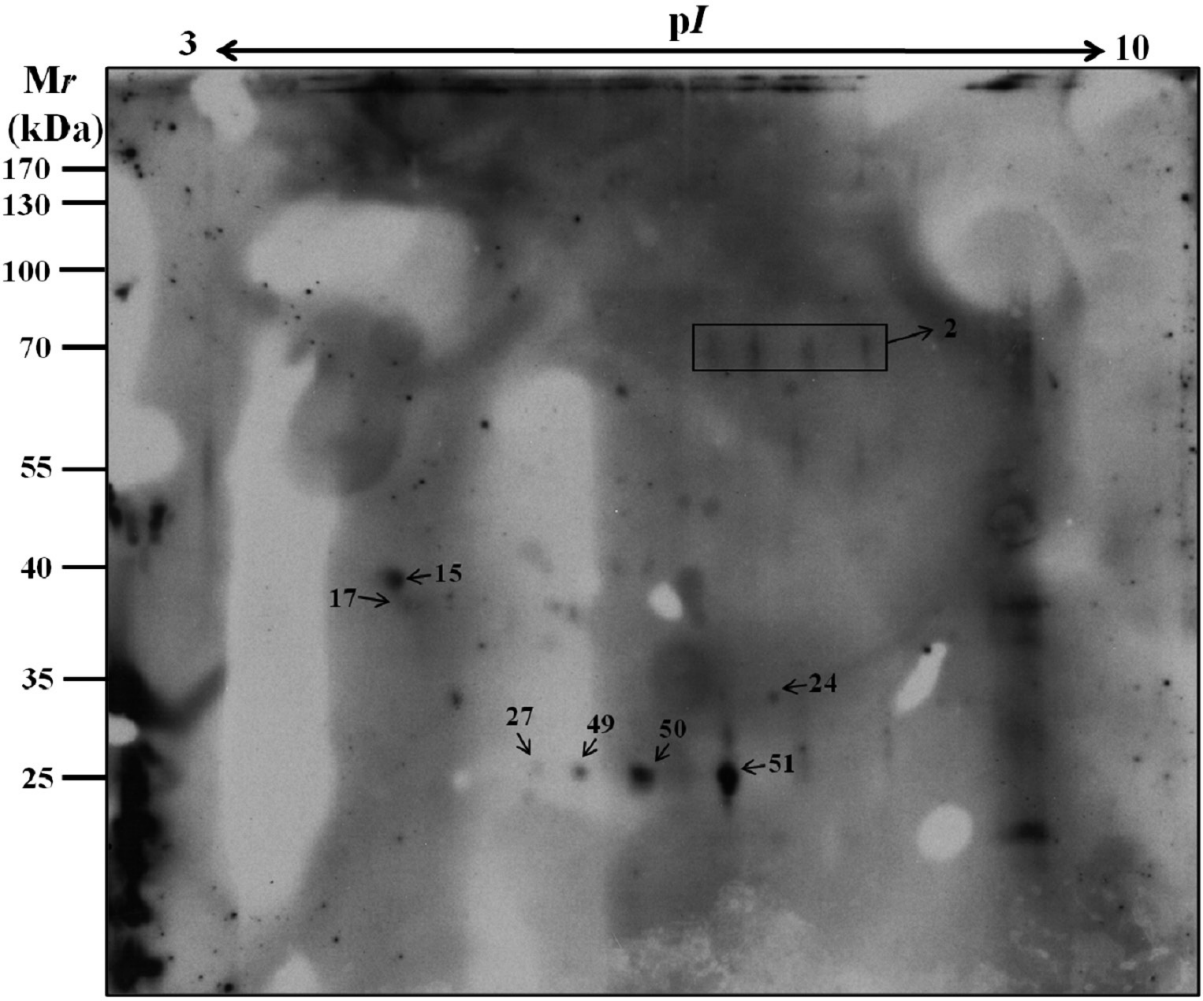
western blotting analysis of eight protein spots from the excretory-secretory products (ESPs) of *Angiostrongylus cantonensis* young adults against serum samples from mice. Eight spots were identified including No.15, No.17, No.24, No.27, No.49, No.50, and No.51.

*Identity of immunoreactive proteins* - In this study, we established the proteome reference map of the ESPs of *A. cantonensis* young adults and used mouse antiserum to detect the immunoreactive proteins by western blotting (Fig. 5). Treatment with uninfected mouse serum and mouse serum-free conditions were used as controls [Supplementary data (Figure)]. A total of 11 protein spots were detected by the serum and were further identified by LC-MS/MS analysis. Approximately eight proteins were identified, including protein disulphide isomerase (*n* = 1), putative aspartic protease (*n* = 1), annexin (*n* = 1), and uncharacterised proteins (*n* = 5) (Table). These identified proteins may be used as potential diagnostic targets for *A. cantonensis* infection.

**TABLE:** The identification of immunoreactive proteins in *Angiostrongylus cantonensis* young adults excretory-secretory products

Spot	Accession	Description
2	A0A158P932	Protein disulfide-isomerase OS=*Angiostrongylus cantonensis* PE=3 SV=1
15	A0A0K0D6W5	Uncharacterised protein OS=*Angiostrongylus cantonensis* PE=4 SV=1
17	R4UYY8	Putative aspartic protease OS=*Angiostrongylus cantonensis* PE=2 SV=1
24	A0A158P8B1	Annexin OS=*Angiostrongylus cantonensis* PE=3 SV=1
27	A0A0K0CU69	Uncharacterised protein OS=*Angiostrongylus cantonensis* PE=4 SV=1
49	A0A0K0CU69	Uncharacterised protein OS=*Angiostrongylus cantonensis* PE=4 SV=1
50	A0A0K0CU69	Uncharacterised protein OS=*Angiostrongylus cantonensis* PE=4 SV=1
51	A0A0K0CU69	Uncharacterised protein OS=*Angiostrongylus cantonensis* PE=4 SV=1

## DISCUSSION

Hosts are infected by ingesting the infective third-stage larvae (L3) of *A. cantonensis* in either an intermediate host (snails) or a paratenic host (freshwater crustaceans and frogs). The young adult worms’ entry into the CNS can induce a series of pathological changes. Our previous studies suggest that rabbits infected with *A. cantonensis* can exhibit pathological changes and neurological abnormalities in brain tissues.^21^ Moreover, treatment with albendazole in rabbits may induce more severe pathological changes; thus, this drug may not be the appropriate treatment for cerebral angiostrongyliasis.^22^ However, *A. cantonensis* infection in mice may cause brain cell death and elevated ROS and antioxidant levels.^20^

The secretion of ESPs from parasitic helminths is important for tissue penetration, larval development, survival, feeding, and regulation of host immune responses.^23^ Therefore, investigation into these ESPs from the young adults may provide further information for the understanding of the invasion and pathogenesis of *A. cantonensis.* In our studies, the ESP from *A. cantonensis* young adults was found to induce oxidative stress and cell apoptosis in astrocytes, but the activation of the Sonic hedgehog (Shh) pathway can reduce cell injury.^15^ We previously demonstrated that cytokine secretion was induced in mouse astrocytes by treatment with ESP of *A. cantonensis* young adult worms.^16^

In recent years, proteomic analysis has become a useful technique for detection and identification of somatic proteins or ESPs from different stages, sexes, hosts, or conditions of parasitic helminths including *A. cantonensis*,^18^ ^,^ ^24^ *Trichinella spiralis*,^25^ ^,^ ^26^ *Dirofilaria immitis*,^27^ *Schistosoma mansoni*,^28^ *Schistosoma japonicum*,^29^ and *Echinococcus granulosus*.^30^ Identification of significant ESP molecules is a good strategy to determine the host-parasite interaction, and may facilitate novel ideas for disease treatment and diagnosis.

Several molecules in the *A. cantonensis* ESPs, such as proteases, are capable of acting as immunoregulators, but the relationship between ESPs and host cell survival is still unknown.^23^ Several proteins were detected in the ESPs of *A. cantonensis* adult worms by reacting with infected patients’ sera.^17^ In the present study, we established the proteome profile of ESPs from young adult worms of *A. cantonensis* and determined the potential immunoreaction properties of the ESPs. Our findings showed that protein disulphide isomerase, calreticulin, peptidyl-prolyl cis-trans isomerase, putative aspartic protease, and galectin were the most abundant proteins. Protein disulphide isomerase has been detected through proteomic analysis in the ESPs of many parasitic worms, such as *A. cantonensis* and *S. mansoni*.^24^ ^,^ ^28^ This protein is a potent oxidoreductase enzyme that catalyses disulphide-dependent conformational folding. Previous studies showed that protein disulphide isomerase is released in the ESPs of schistosomes, and the protein was found to play an important role in the modulation of host immune responses.^31^ Furthermore, protein disulphide isomerase may regulate survival and virulence in *Leishmania major*.^32^ Calreticulin widely exists in eukaryotic cells, and this protein can regulate gene transcription, protein folding, endoplasmic reticulum stress, and calcium concentration in cells.^33^ ^,^ ^34^ Some proteomic studies detected calreticulin in *T. spiralis*, *E. granulosus*, and *D. immitis*.^27^ ^,^ ^30^ ^,^ ^35^ In parasitic helminths, proteases play an important role in survival and development, including molting, protein digestion, migration, and regulation of host immune responses.^36^ ^,^ ^37^ Aspartic proteases could be used to digest the host haemoglobin in parasitic nematodes, such as *Brugia malayi*,^38^ *T. spiralis*,^39^ ^,^ ^40^ and *Steinernema carpocapsae*.^41^ In our study, putative aspartic proteases were found to be secreted from *A. cantonensis* young adults and detected by 2-DE and LC-MS/MS analysis.

In a previous study, we used proteomic analysis to establish the reference map for somatic proteins of young adults of *A. cantonensis* and found that HSP60 was the most highly expressed in the body of the worm.^18^ However, this study showed that the most abundant proteins in the ESPs of young adults were disulphide isomerase and calreticulin. Therefore, the component proteins of ESPs and somatic proteins are extremely different. This finding will be useful for subsequent research on host-parasite interaction and nematodes pathogenicity in *A. cantonensis* infection.

A suspected *A. cantonensis* infection can be confirmed only by detection of *A. cantonensis-*specific antibodies via an enzyme-linked immunosorbent assay (ELISA) with the host serum or by identification of the young adults in the cerebrospinal fluid (CSF). Currently, anthelmintic drugs such as albendazole or mebendazole are used for the clinical treatment of angiostrongyliasis. However, results of albendazole treatment showed that pathological changes are more severe in the brain. These results suggest that albendazole treatment may not be appropriate for cerebral angiostrongyliasis.^22^ In this study, we used proteomic analysis to determine the components of ESPs from *A. cantonensis* young adults; then, we detected the proteins in the ESPs that are immunoreactive with the serum of *A. cantonensis*-infected mouse via western blotting. These immunoreactive proteins (protein disulphide isomerase, putative aspartic protease, and annexin) may be helpful for angiostrongyliasis diagnosis and treatment.
