# Supplementary material for: Proteomic analysis of excretory-secretory products from young adults of Angiostrongylus cantonensis
Source: Mem Inst Oswaldo Cruz. 2019 Jun 19;114:e180556. doi: 10.1590/0074-02760180556 (PMC6594673; doi:10.1590/0074-02760180556)
Supplement: Supplementary file 1 [file 1678-8060-mioc-114-e180556-s.pdf]

TABLE I

Identification of *Angiostrongylus cantonensis* excretory-secretory products (ESPs) in the two-dimensional gel electrophoresis (2-DE) reference map

Spot	Accession	Description (UniProt)	Area	Score	# AAs	MW [kDa]
1	A0A158P600	Uncharactersed protein OS=*Angiostrongylus cantonensis* PE=4 SV=1 - [A0A158P600_ANGCA]	1,107E8	1098,96	528	59,3
2	A0A158P932	Protein disulfide-isomerase OS=*Angiostrongylus cantonensis* PE=3 SV=1 - [A0A158P932_ANGCA]	4,667E6	70,70	485	54,5
3	A0A158PB90	Uncharactersed protein OS=*Angiostrongylus cantonensis* PE=3 SV=1 - [A0A158PB90_ANGCA]	5,654E5	68,61	506	55,8
4	A0A158PB90	Uncharactersed protein OS=*Angiostrongylus cantonensis* PE=3 SV=1 - [A0A158PB90_ANGCA]	1,829E8	1348,60	506	55,8
5	A0A158P932	Protein disulfide-isomerase OS=*Angiostrongylus cantonensis* PE=3 SV=1 - [A0A158P932_ANGCA]	1,074E9	5417,73	485	54,5
6	A0A0K0D370	Protein disulfide-isomerase OS=*Angiostrongylus cantonensis* PE=3 SV=1 - [A0A0K0D370_ANGCA]	2,555E9	6585,78	465	51,9
7	G0YXZ1	Calreticulin OS=*Angiostrongylus cantonensis* GN=crt PE=2 SV=1 - [G0YXZ1_ANGCA]	1,232E9	2241,16	401	46,5
8	A0A158P932	Protein disulfide-isomerase OS=*Angiostrongylus cantonensis* PE=3 SV=1 - [A0A158P932_ANGCA]	1,600E7	329,71	485	54,5
9	A0A158P5R7	Uncharactersed protein OS=*Angiostrongylus cantonensis* PE=4 SV=1 - [A0A158P5R7_ANGCA]	1,253E8	871,17	416	45,8
10	G0YXZ1	Calreticulin OS=*Angiostrongylus cantonensis* GN=crt PE=2 SV=1 - [G0YXZ1_ANGCA]	7,395E7	402,03	401	46,5
11	A0A0K0DGK5	Uncharactersed protein OS=*Angiostrongylus cantonensis* PE=4 SV=1 - [A0A0K0DGK5_ANGCA]	6,522E7	210,24	252	28,2
12	R4UYY8	Putative aspartic protease OS=*Angiostrongylus cantonensis* PE=2 SV=1 - [R4UYY8_ANGCA]	1,254E7	422,57	425	46,2
13	A0A0K0DPT1	Uncharactersed protein OS=*Angiostrongylus cantonensis* PE=3 SV=1 - [A0A0K0DPT1_ANGCA]	2,213E9	4830,10	376	41,8
14	A0A158P932	Protein disulfide-isomerase OS=*Angiostrongylus cantonensis* PE=3 SV=1 - [A0A158P932_ANGCA]	4,132E7	741,88	485	54,5
15	A0A0K0D6W5	Uncharactersed protein OS=*Angiostrongylus cantonensis* PE=4 SV=1 - [A0A0K0D6W5_ANGCA]	4,836E7	267,77	68	7,7
16	R4UYY8	Putative aspartic protease OS=*Angiostrongylus cantonensis* PE=2 SV=1 - [R4UYY8_ANGCA]	5,004E6	333,83	425	46,2
17	R4UYY8	Putative aspartic protease OS=*Angiostrongylus cantonensis* PE=2 SV=1 - [R4UYY8_ANGCA]	3,243E7	559,74	425	46,2
18	A0A0K0D370	Protein disulfide-isomerase OS=*Angiostrongylus cantonensis* PE=3 SV=1 - [A0A0K0D370_ANGCA]	2,959E8	2210,75	465	51,9
19	A0A0K0D370	Protein disulfide-isomerase OS=*Angiostrongylus cantonensis* PE=3 SV=1 - [A0A0K0D370_ANGCA]	2,791E8	2361,30	465	51,9
20	A0A0K0D370	Protein disulfide-isomerase OS=*Angiostrongylus cantonensis* PE=3 SV=1 - [A0A0K0D370_ANGCA]	2,795E8	1378,73	465	51,9
21	G1EUS5	Galectin OS=*Angiostrongylus cantonensis* PE=2 SV=1 - [G1EUS5_ANGCA]	4,475E7	636,26	316	36,0
22	G1EUS5	Galectin OS=*Angiostrongylus cantonensis* PE=2 SV=1 - [G1EUS5_ANGCA]	6,182E8	1151,98	316	36,0
23	A0A0K0DHN4	Transaldolase OS=*Angiostrongylus cantonensis* PE=3 SV=1 - [A0A0K0DHN4_ANGCA]	3,489E6	363,46	321	36,0
24	A0A158P8B1	Annexin OS=*Angiostrongylus cantonensis* PE=3 SV=1 - [A0A158P8B1_ANGCA]	2,020E9	1552,97	320	36,0
25	G1EUS8	NAC domain containing protein OS=*Angiostrongylus cantonensis* PE=2 SV=1 - [G1EUS8_ANGCA]	1,660E6	131,60	185	20,4
26	A0A0K0D9J3	Uncharactersed protein OS=*Angiostrongylus cantonensis* PE=4 SV=1 - [A0A0K0D9J3_ANGCA]	2,262E8	666,36	252	27,9
27	A0A0K0CU69	Uncharactersed protein OS=*Angiostrongylus cantonensis* PE=4 SV=1 - [A0A0K0CU69_ANGCA]	6,539E7	895,58	260	28,6
28	A0A0K0CU69	Uncharactersed protein OS=*Angiostrongylus cantonensis* PE=4 SV=1 - [A0A0K0CU69_ANGCA]	2,116E7	762,70	260	28,6
29	A0A0K0CVZ7	Uncharactersed protein OS=*Angiostrongylus cantonensis* PE=4 SV=1 - [A0A0K0CVZ7_ANGCA]	2,164E8	516,74	228	25,8
30	G0YXZ2	14-3-3 protein OS=*Angiostrongylus cantonensis* PE=2 SV=1 - [G0YXZ2_ANGCA]	1,393E9	2858,34	251	28,5
31	A0A0K0CVZ7	Uncharactersed protein OS=*Angiostrongylus cantonensis* PE=4 SV=1 - [A0A0K0CVZ7_ANGCA]	8,170E8	2103,04	228	25,8

Spot	Accession	Description (UniProt)	Area	Score	# AAs	MW [kDa]
32	A0A158PB99	Uncharactersed protein OS=*Angiostrongylus cantonensis* PE=3 SV=1 - [A0A158PB99_ANGCA]	3,299E8	1063,74	395	44,5
33	A0A158PB99	Uncharactersed protein OS=*Angiostrongylus cantonensis* PE=3 SV=1 - [A0A158PB99_ANGCA]	4,115E8	886,24	395	44,5
34	A0A0K0DKE8	Uncharactersed protein OS=*Angiostrongylus cantonensis* PE=4 SV=1 - [A0A0K0DKE8_ANGCA]	6,872E7	695,70	210	22,8
35	A0A0K0D5E2	Uncharactersed protein OS=*Angiostrongylus cantonensis* PE=4 SV=1 - [A0A0K0D5E2_ANGCA]	1,377E9	781,25	132	14,8
36	A0A0K0DHF4	Peptidyl-prolyl cis-trans isomerase OS=*Angiostrongylus cantonensis* PE=3 SV=1 - [A0A0K0DHF4_ANGCA]	3,037E7	168,74	163	17,5
37	A0A0K0D5E2	Uncharactersed protein OS=*Angiostrongylus cantonensis* PE=4 SV=1 - [A0A0K0D5E2_ANGCA]	5,711E8	623,49	132	14,8
38	A0A0K0CWI0	Uncharactersed protein OS=*Angiostrongylus cantonensis* PE=4 SV=1 - [A0A0K0CWI0_ANGCA]	2,272E9	4561,24	203	22,7
39	A0A0K0CWI0	Uncharactersed protein OS=*Angiostrongylus cantonensis* PE=4 SV=1 - [A0A0K0CWI0_ANGCA]	3,192E9	5594,41	203	22,7
40	A0A0K0CWI0	Uncharactersed protein OS=*Angiostrongylus cantonensis* PE=4 SV=1 - [A0A0K0CWI0_ANGCA]	3,793E9	4012,08	203	22,7
41	A0A0K0D5E2	Uncharactersed protein OS=*Angiostrongylus cantonensis* PE=4 SV=1 - [A0A0K0D5E2_ANGCA]	1,744E8	125,78	132	14,8
42	A0A0K0D2A0	Peptidyl-prolyl cis-trans isomerase OS=*Angiostrongylus cantonensis* PE=3 SV=1 - [A0A0K0D2A0_ANGCA]	4,427E9	3065,86	178	19,6
43	A0A0K0CWI0	Uncharactersed protein OS=*Angiostrongylus cantonensis* PE=4 SV=1 - [A0A0K0CWI0_ANGCA]	4,115E8	365,14	203	22,7
44	A0A0K0CZS1	Uncharactersed protein OS=*Angiostrongylus cantonensis* PE=3 SV=1 - [A0A0K0CZS1_ANGCA]	6,201E8	652,32	301	33,5
45	A0A0K0DQR1	Peptidyl-prolyl cis-trans isomerase OS=*Angiostrongylus cantonensis* PE=3 SV=1 - [A0A0K0DQR1_ANGCA]	3,841E8	859,13	159	17,2
46	A0A0K0D9J3	Uncharactersed protein OS=*Angiostrongylus cantonensis* PE=4 SV=1 - [A0A0K0D9J3_ANGCA]	9,077E8	854,98	252	27,9
47	A0A0K0DLC2	Uncharactersed protein OS=*Angiostrongylus cantonensis* PE=4 SV=1 - [A0A0K0DLC2_ANGCA]	8,130E6	306,31	209	22,2
48	A0A0K0CU69	Uncharactersed protein OS=*Angiostrongylus cantonensis* PE=4 SV=1 - [A0A0K0CU69_ANGCA]	6,406E7	766,05	260	28,6
49	A0A0K0CU69	Uncharactersed protein OS=*Angiostrongylus cantonensis* PE=4 SV=1 - [A0A0K0CU69_ANGCA]	2,207E8	1208,97	260	28,6
50	A0A0K0CU69	Uncharactersed protein OS=*Angiostrongylus cantonensis* PE=4 SV=1 - [A0A0K0CU69_ANGCA]	6,825E8	3038,64	260	28,6
51	A0A0K0CU69	Uncharactersed protein OS=*Angiostrongylus cantonensis* PE=4 SV=1 - [A0A0K0CU69_ANGCA]	4,563E8	1240,00	260	28,6

Description (UniProt and NCBI)

Spot	Description (UniProt)	Description (NCBI)
1	Uncharacterised protein OS=*Angiostrongylus cantonensis* PE=4 SV=1 - [A0A158P600_ANGCA]	Alpha-2-macroglobulin family protein (*Teladorsagia circumcincta*) [A0A2G9UXM6] 94.6%
2	Protein disulfide-isomerase OS=*Angiostrongylus cantonensis* PE=3 SV=1 - [A0A158P932_ANGCA]	Protein disulfide-isomerase (*Angiostrongylus cantonensis*) [A0A158P932] 100.0%
3	Uncharactersed protein OS=*Angiostrongylus cantonensis* PE=3 SV=1 - [A0A158PB90_ANGCA]	2,3-bisphosphoglycerate-independent phosphoglycerate mutase (*Dictyocaulus viviparus*) [A0A0D8XT87] 81.6%
4	Uncharactersed protein OS=*Angiostrongylus cantonensis* PE=3 SV=1 - [A0A158PB90_ANGCA]	2,3-bisphosphoglycerate-independent phosphoglycerate mutase (*Dictyocaulus viviparus*) [A0A0D8XT87] 81.6%
5	Protein disulfide-isomerase OS=*Angiostrongylus cantonensis* PE=3 SV=1 - [A0A158P932_ANGCA]	Protein disulfide-isomerase (*Angiostrongylus cantonensis*) [A0A158P932] 100.0%
6	Protein disulfide-isomerase OS=*Angiostrongylus cantonensis* PE=3 SV=1 - [A0A0K0D370_ANGCA]	Protein disulfide-isomerase (*Angiostrongylus cantonensis*) [A0A0K0D370] 100.0%
7	Calreticulin OS=*Angiostrongylus cantonensis* GN=crt PE=2 SV=1 - [G0YXZ1_ANGCA]	Calreticulin (*Angiostrongylus cantonensis*) [G0YXZ1] 100.0%
8	Protein disulfide-isomerase OS=*Angiostrongylus cantonensis* PE=3 SV=1 - [A0A158P932_ANGCA]	Protein disulfide-isomerase (*Angiostrongylus cantonensis*) [A0A158P932] 100.0%
9	Uncharactersed protein OS=*Angiostrongylus cantonensis* PE=4 SV=1 - [A0A158P5R7_ANGCA]	Fumarylacetoacetase family protein (*Ancylostoma caninum*) [A0A368GH57] 87.5%
10	Calreticulin OS=*Angiostrongylus cantonensis* GN=crt PE=2 SV=1 - [G0YXZ1_ANGCA]	Calreticulin (*Angiostrongylus cantonensis*) [G0YXZ1] 100.0%

Spot	Description (UniProt)	Description (NCBI)
11	Uncharactersed protein OS=*Angiostrongylus cantonensis* PE=4 SV=1 - [A0A0K0DGK5_ANGCA]	Kunitz/Bovine pancreatic trypsin inhibitor domain protein (*Ancylostoma caninum*) [A0A368H1Y6] 54.4%
12	Putative aspartic protease OS=*Angiostrongylus cantonensis* PE=2 SV=1 - [R4UYY8_ANGCA]	Putative aspartic protease (*Angiostrongylus cantonensis*) [AGM38618] 100.0%
13	Uncharactersed protein OS=*Angiostrongylus cantonensis* PE=3 SV=1 - [A0A0K0DPT1_ANGCA]	Actin (*Ancylostoma caninum*) [A0A368FUA3] 100%
14	Protein disulfide-isomerase OS=*Angiostrongylus cantonensis* PE=3 SV=1 - [A0A158P932_ANGCA]	Protein disulfide-isomerase (*Angiostrongylus cantonensis*) [A0A158P932] 100.0%
15	Uncharactersed protein OS=*Angiostrongylus cantonensis* PE=4 SV=1 - [A0A0K0D6W5_ANGCA]	EF hand (*Necator americanus*) [W2TTV2] 85.4%
16	Putative aspartic protease OS=*Angiostrongylus cantonensis* PE=2 SV=1 - [R4UYY8_ANGCA]	Putative aspartic protease (*Angiostrongylus cantonensis*) [AGM38618] 100.0%
17	Putative aspartic protease OS=*Angiostrongylus cantonensis* PE=2 SV=1 - [R4UYY8_ANGCA]	Putative aspartic protease (*Angiostrongylus cantonensis*) [AGM38618] 100.0%
18	Protein disulfide-isomerase OS=*Angiostrongylus cantonensis* PE=3 SV=1 - [A0A0K0D370_ANGCA]	Protein disulfide-isomerase (*Angiostrongylus cantonensis*) [A0A0K0D370] 100.0%
19	Protein disulfide-isomerase OS=*Angiostrongylus cantonensis* PE=3 SV=1 - [A0A0K0D370_ANGCA]	Protein disulfide-isomerase (*Angiostrongylus cantonensis*) [A0A0K0D370] 100.0%
20	Protein disulfide-isomerase OS=*Angiostrongylus cantonensis* PE=3 SV=1 - [A0A0K0D370_ANGCA]	Protein disulfide-isomerase (*Angiostrongylus cantonensis*) [A0A0K0D370] 100.0%
21	Galectin OS=*Angiostrongylus cantonensis* PE=2 SV=1 - [G1EUS5_ANGCA]	Galectin (*Angiostrongylus cantonensis*) [G1EUS5] 100%
22	Galectin OS=*Angiostrongylus cantonensis* PE=2 SV=1 - [G1EUS5_ANGCA]	Galectin (*Angiostrongylus cantonensis*) [G1EUS5] 100%
23	Transaldolase OS=*Angiostrongylus cantonensis* PE=3 SV=1 - [A0A0K0DHN4_ANGCA]	Transaldolase (*Angiostrongylus cantonensis*) [A0A0K0DHN4] 100%
24	Annexin OS=*Angiostrongylus cantonensis* PE=3 SV=1 - [A0A158P8B1_ANGCA]	Annexin (*Angiostrongylus cantonensis*) [A0A158P8B1] 100%
25	NAC domain containing protein OS=*Angiostrongylus cantonensis* PE=2 SV=1 - [G1EUS8_ANGCA]	NAC domain containing protein (*Angiostrongylus cantonensis*) [G1EUS8] 100%
26	Uncharactersed protein OS=*Angiostrongylus cantonensis* PE=4 SV=1 - [A0A0K0D9J3_ANGCA]	Aspartyl protease inhibitor (*Angiostrongylus cantonensis*) [A0A1L6PVG8] 100%
27	Uncharactersed protein OS=*Angiostrongylus cantonensis* PE=4 SV=1 - [A0A0K0CU69_ANGCA]	Sushi domain protein (*Oesophagostomum dentatum*) [A0A0B1T3J6] 26.4%
28	Uncharactersed protein OS=*Angiostrongylus cantonensis* PE=4 SV=1 - [A0A0K0CU69_ANGCA]	Sushi domain protein (*Oesophagostomum dentatum*) [A0A0B1T3J6] 26.4%
29	Uncharactersed protein OS=*Angiostrongylus cantonensis* PE=4 SV=1 - [A0A0K0CVZ7_ANGCA]	Putative major allergen (*Angiostrongylus cantonensis*) [C7BVN8] 41.6%
30	14-3-3 protein OS=*Angiostrongylus cantonensis* PE=2 SV=1 - [G0YXZ2_ANGCA]	14-3-3 protein (*Angiostrongylus cantonensis*) [G0YXZ2] 100%
31	Uncharactersed protein OS=*Angiostrongylus cantonensis* PE=4 SV=1 - [A0A0K0CVZ7_ANGCA]	Putative major allergen (*Angiostrongylus cantonensis*) [C7BVN8] 41.6%
32	Uncharactersed protein OS=*Angiostrongylus cantonensis* PE=3 SV=1 - [A0A158PB99_ANGCA]	Triosephosphate isomerase (*Angiostrongylus cantonensis*) [A0A158PB99] 100%
33	Uncharactersed protein OS=*Angiostrongylus cantonensis* PE=3 SV=1 - [A0A158PB99_ANGCA]	Triosephosphate isomerase (*Angiostrongylus cantonensis*) [A0A158PB99] 100%
34	Uncharactersed protein OS=*Angiostrongylus cantonensis* PE=4 SV=1 - [A0A0K0DKE8_ANGCA]	Putative acyl-protein thioesterase 1 (*Dictyocaulus viviparus*) [A0A0D8Y6A4] 89.8%
35	Uncharactersed protein OS=*Angiostrongylus cantonensis* PE=4 SV=1 - [A0A0K0D5E2_ANGCA]	Glutathione S-transferase 2 (*Heligmosomoides polygyrus*) [Q9NJQ6] 84.8%
36	Peptidyl-prolyl cis-trans isomerase OS=*Angiostrongylus cantonensis* PE=3 SV=1 - [A0A0K0DHF4_ANGCA]	Peptidyl-prolyl cis-trans isomerase (*Angiostrongylus cantonensis*) [A0A0K0DHF4] 100%
37	Uncharactersed protein OS=*Angiostrongylus cantonensis* PE=4 SV=1 - [A0A0K0D5E2_ANGCA]	Glutathione S-transferase 2 (*Heligmosomoides polygyrus*) [Q9NJQ6] 84.8%
38	Uncharactersed protein OS=*Angiostrongylus cantonensis* PE=4 SV=1 - [A0A0K0CWI0_ANGCA]	Antioxidant, AhpC/TSA family (*Dictyocaulus viviparus*)[A0A0D8Y3P4] 91.2%
39	Uncharacterised protein OS=*Angiostrongylus cantonensis* PE=4 SV=1 - [A0A0K0CWI0_ANGCA]	Antioxidant, AhpC/TSA family (*Dictyocaulus viviparus*)[A0A0D8Y3P4] 91.2%
40	Uncharacterised protein OS=*Angiostrongylus cantonensis* PE=4 SV=1 - [A0A0K0CWI0_ANGCA]	Antioxidant, AhpC/TSA family (*Dictyocaulus viviparus*)[A0A0D8Y3P4] 91.2%
41	Uncharacterised protein OS=*Angiostrongylus cantonensis* PE=4 SV=1 - [A0A0K0D5E2_ANGCA]	Glutathione S-transferase 2 (*Heligmosomoides polygyrus*) [Q9NJQ6] 84.8%
42	Peptidyl-prolyl cis-trans isomerase OS=*Angiostrongylus cantonensis* PE=3 SV=1 - [A0A0K0D2A0_ANGCA]	Peptidyl-prolyl cis-trans isomerase (=*Angiostrongylus cantonensis*) [A0A0K0D2A0] 100%
43	Uncharacterised protein OS=*Angiostrongylus cantonensis* PE=4 SV=1 - [A0A0K0CWI0_ANGCA]	Antioxidant, AhpC/TSA family (*Dictyocaulus viviparus*)[A0A0D8Y3P4] 91.2%

Spot	Description (UniProt)	Description (NCBI)
44	Uncharacterised protein OS=*Angiostrongylus cantonensis* PE=3 SV=1 - [A0A0K0CsS1_ANGCA]	Protein CBR-UNC-60 (*Caenorhabditis briggsae*) [A8X2K2] 84.5%
45	Peptidyl-prolyl cis-trans isomerase OS=*Angiostrongylus cantonensis* PE=3 SV=1 - [A0A0K0DQR1_ANGCA]	Peptidyl-prolyl cis-trans isomerase (=*Angiostrongylus cantonensis*) [A0A0K0D2A0] 100%
46	Uncharacterised protein OS=*Angiostrongylus cantonensis* PE=4 SV=1 - [A0A0K0D9J3_ANGCA]	Aspartyl protease inhibitor (*Angiostrongylus cantonensis*) [A0A1L6PVG8] 100%
47	Uncharacterised protein OS=*Angiostrongylus cantonensis* PE=4 SV=1 - [A0A0K0DLC2_ANGCA]	Uncharacterised protein(*Angiostrongylus cantonensis*) [A0A0K0DLC2] 100%
48	Uncharacterised protein OS=*Angiostrongylus cantonensis* PE=4 SV=1 - [A0A0K0CU69_ANGCA]	Sushi domain protein (*Oesophagostomum dentatum*) [A0A0B1T3J6] 26.4%
49	Uncharacterised protein OS=*Angiostrongylus cantonensis* PE=4 SV=1 - [A0A0K0CU69_ANGCA]	Sushi domain protein (*Oesophagostomum dentatum*) [A0A0B1T3J6] 26.4%
50	Uncharacterised protein OS=*Angiostrongylus cantonensis* PE=4 SV=1 - [A0A0K0CU69_ANGCA]	Sushi domain protein (*Oesophagostomum dentatum*) [A0A0B1T3J6] 26.4%
51	Uncharacterised protein OS=*Angiostrongylus cantonensis* PE=4 SV=1 - [A0A0K0CU69_ANGCA]	Sushi domain protein (*Oesophagostomum dentatum*) [A0A0B1T3J6] 26.4%

TABLE II

Identification of *Angiostrongylus cantonensis* excretory-secretory products (ESPs) by liquid chromatography-mass spectrometry (LC-MS/MS)

Accession	Description	Area	Score	# AAs	MW [kDa]	Relative protein abundance [%]
A0A0K0DR90	Uncharacterised protein OS=*Angiostrongylus cantonensis* PE=4 SV=1 - [A0A0K0DR90_ANGCA]	1,611E9	10671,71	897	103,4	9,67%
A0A0K0DRF2	Uncharacterised protein OS=*Angiostrongylus cantonensis* PE=4 SV=1 - [A0A0K0DRF2_ANGCA]	4,845E8	3453,37	446	52,6	3,74%
A0A0K0DH49	Uncharacterised protein OS=*Angiostrongylus cantonensis* PE=4 SV=1 - [A0A0K0DH49_ANGCA]	2,458E9	3149,28	149	16,1	2,38%
C7BVM2	Putative Lipid Binding Protein OS=*Angiostrongylus cantonensis* GN=lbp-1 PE=2 SV=1 - [C7BVM2_ANGCA]	2,666E8	1969,53	163	19,0	1,95%
A0A158P932	Protein disulfide-isomerase OS=*Angiostrongylus cantonensis* PE=3 SV=1 - [A0A158P932_ANGCA]	4,112E8	1857,56	485	54,5	1,76%
A0A0K0D370	Protein disulfide-isomerase OS=*Angiostrongylus cantonensis* PE=3 SV=1 - [A0A0K0D370_ANGCA]	1,767E8	2224,64	465	51,9	1,74%
A0A0K0DR15	Uncharacterised protein OS=*Angiostrongylus cantonensis* PE=4 SV=1 - [A0A0K0DR15_ANGCA]	5,792E8	1567,04	180	21,0	1,72%
A0A0K0DID8	Uncharacterised protein OS=*Angiostrongylus cantonensis* PE=4 SV=1 - [A0A0K0DID8_ANGCA]	7,473E8	2117,07	205	22,4	1,52%
A0A158P7K4	Uncharacterised protein OS=*Angiostrongylus cantonensis* PE=4 SV=1 - [A0A158P7K4_ANGCA]	7,390E7	1795,06	358	39,8	1,52%
A0A0K0DPT1	Uncharacterised protein OS=*Angiostrongylus cantonensis* PE=3 SV=1 - [A0A0K0DPT1_ANGCA]	3,444E8	1475,05	376	41,8	1,36%
A0A0K0D9I3	Uncharacterised protein OS=*Angiostrongylus cantonensis* PE=3 SV=1 - [A0A0K0D9I3_ANGCA]	2,654E8	1425,44	376	41,8	1,29%
G0YXZ2	14-3-3 protein OS=*Angiostrongylus cantonensis* PE=2 SV=1 - [G0YXZ2_ANGCA]	1,298E8	1371,18	251	28,5	1,24%
G1EUS5	Galectin OS=*Angiostrongylus cantonensis* PE=2 SV=1 - [G1EUS5_ANGCA]	8,637E7	1175,67	316	36,0	1,24%
A0A0K0CY69	Uncharacterised protein OS=*Angiostrongylus cantonensis* PE=3 SV=1 - [A0A0K0CY69_ANGCA]	4,759E7	1391,33	659	72,2	1,17%
A0A158P6Y9	Uncharacterised protein OS=*Angiostrongylus cantonensis* PE=3 SV=1 - [A0A158P6Y9_ANGCA]	3,444E8	1278,01	376	41,8	1,12%
A0A0K0CU69	Uncharacterised protein OS=*Angiostrongylus cantonensis* PE=4 SV=1 - [A0A0K0CU69_ANGCA]	1,262E8	1544,94	260	28,6	1,12%
A0A0K0DBN3	Cystatin OS=*Angiostrongylus cantonensis* PE=3 SV=1 - [A0A0K0DBN3_ANGCA]	5,324E8	1495,77	144	15,8	1,02%
A0A0K0D9J3	Uncharacterised protein OS=*Angiostrongylus cantonensis* PE=4 SV=1 - [A0A0K0D9J3_ANGCA]	5,053E8	1258,86	252	27,9	1,02%
C7BVR7	Putative Conserved Cystein/Glycine domain protein OS=*Angiostrongylus cantonensis* GN=ccg-1 PE=2 SV=1 - [C7BVR7_ANGCA]	4,026E8	1027,34	292	32,6	0,95%
A0A0K0CUV9	Uncharacterised protein OS=*Angiostrongylus cantonensis* PE=3 SV=1 - [A0A0K0CUV9_ANGCA]	4,448E7	1622,40	434	47,2	0,95%
A0A0K0CVJ3	Uncharacterised protein OS=*Angiostrongylus cantonensis* PE=3 SV=1 - [A0A0K0CVJ3_ANGCA]	4,365E7	1133,22	707	81,2	0,95%
A0A0K0DEG4	Uncharacterised protein OS=*Angiostrongylus cantonensis* PE=4 SV=1 - [A0A0K0DEG4_ANGCA]	1,900E8	976,25	136	15,0	0,95%
A0A0K0CZI7	Uncharacterised protein OS=*Angiostrongylus cantonensis* PE=3 SV=1 - [A0A0K0CZI7_ANGCA]	2,959E7	1549,91	740	84,9	0,91%
A0A158P8B1	Annexin OS=*Angiostrongylus cantonensis* PE=3 SV=1 - [A0A158P8B1_ANGCA]	2,532E7	1191,55	320	36,0	0,88%
A0A0K0DII7	4-hydroxyphenylpyruvate dioxygenase OS=*Angiostrongylus cantonensis* PE=3 SV=1 - [A0A0K0DII7_ANGCA]	4,618E7	880,38	366	41,4	0,86%
A0A158PB90	Uncharacterised protein OS=*Angiostrongylus cantonensis* PE=3 SV=1 - [A0A158PB90_ANGCA]	2,691E7	860,41	506	55,8	0,86%
A0A158PBY8	Uncharacterised protein OS=*Angiostrongylus cantonensis* PE=4 SV=1 - [A0A158PBY8_ANGCA]	3,755E8	1327,22	239	27,7	0,83%
A0A0K0DGX0	Fructose-bisphosphate aldolase OS=*Angiostrongylus cantonensis* PE=3 SV=1 - [A0A0K0DGX0_ANGCA]	3,388E7	1123,01	674	72,7	0,81%
A0A158P9C1	Uncharacterised protein OS=*Angiostrongylus cantonensis* PE=4 SV=1 - [A0A158P9C1_ANGCA]	1,456E7	847,68	1587	175,0	0,81%
A0A0K0DDS4	Fructose-bisphosphate aldolase OS=*Angiostrongylus cantonensis* PE=3 SV=1 - [A0A0K0DDS4_ANGCA]	5,276E7	973,14	366	39,3	0,79%

Accession	Description	Area	Score	# AAs	MW [kDa]	Relative protein abundance [%]
A0A0K0CX61	Uncharacterised protein OS=*Angiostrongylus cantonensis* PE=4 SV=1 - [A0A0K0CX61_ANGCA]	1,951E7	1079,88	355	37,6	0,79%
A0A0K0CZV4	Uncharacterised protein OS=*Angiostrongylus cantonensis* PE=4 SV=1 - [A0A0K0CZV4_ANGCA]	1,712E7	733,14	852	94,6	0,79%
A0A0K0DC78	Uncharacterised protein OS=*Angiostrongylus cantonensis* PE=4 SV=1 - [A0A0K0DC78_ANGCA]	4,394E7	766,23	542	60,8	0,79%
A0A0K0DQQ3	Uncharacterised protein OS=*Angiostrongylus cantonensis* PE=3 SV=1 - [A0A0K0DQQ3_ANGCA]	2,760E7	761,58	404	45,7	0,74%
A0A0K0CWI0	Uncharacterised protein OS=*Angiostrongylus cantonensis* PE=4 SV=1 - [A0A0K0CWI0_ANGCA]	1,379E8	690,60	203	22,7	0,74%
A0A0K0CXZ9	Uncharacterised protein OS=*Angiostrongylus cantonensis* PE=4 SV=1 - [A0A0K0CXZ9_ANGCA]	1,039E8	987,17	126	14,1	0,74%
A0A0K0CYV3	Uncharacterised protein OS=*Angiostrongylus cantonensis* PE=4 SV=1 - [A0A0K0CYV3_ANGCA]	1,323E8	955,26	139	15,1	0,74%
A0A0K0D1S1	Uncharacterised protein OS=*Angiostrongylus cantonensis* PE=4 SV=1 - [A0A0K0D1S1_ANGCA]	2,545E8	791,32	154	17,4	0,74%
A0A0K0DLC2	Uncharacterised protein OS=*Angiostrongylus cantonensis* PE=4 SV=1 - [A0A0K0DLC2_ANGCA]	1,098E8	1258,72	209	22,2	0,71%
A0A0K0CWC8	Uncharacterised protein OS=*Angiostrongylus cantonensis* PE=4 SV=1 - [A0A0K0CWC8_ANGCA]	2,843E8	882,69	198	21,8	0,69%
A0A0K0CYS1	Uncharacterised protein OS=*Angiostrongylus cantonensis* PE=4 SV=1 - [A0A0K0CYS1_ANGCA]	7,872E7	534,18	146	16,6	0,69%
A0A0K0DMW3	Uncharacterised protein OS=*Angiostrongylus cantonensis* PE=3 SV=1 - [A0A0K0DMW3_ANGCA]	4,421E7	657,78	312	35,8	0,67%
A0A0K0CST2	Uncharacterised protein OS=*Angiostrongylus cantonensis* PE=4 SV=1 - [A0A0K0CST2_ANGCA]	2,250E7	659,58	448	50,3	0,67%
A0A0K0CVZ7	Uncharacterised protein OS=*Angiostrongylus cantonensis* PE=4 SV=1 - [A0A0K0CVZ7_ANGCA]	2,288E8	771,90	228	25,8	0,67%
A0A158PCC1	Uncharacterised protein OS=*Angiostrongylus cantonensis* PE=4 SV=1 - [A0A158PCC1_ANGCA]	2,441E8	919,61	140	15,5	0,64%
A0A0K0D741	Uncharacterised protein OS=*Angiostrongylus cantonensis* PE=3 SV=1 - [A0A0K0D741_ANGCA]	2,303E7	517,26	163	18,9	0,62%
A0A0K0CZS1	Uncharacterised protein OS=*Angiostrongylus cantonensis* PE=3 SV=1 - [A0A0K0CZS1_ANGCA]	1,467E7	599,55	301	33,5	0,60%
A0A0K0CWR1	Uncharacterised protein OS=*Angiostrongylus cantonensis* PE=4 SV=1 - [A0A0K0CWR1_ANGCA]	1,234E8	599,42	215	23,9	0,60%
A0A0K0D3U9	Uncharacterised protein OS=*Angiostrongylus cantonensis* PE=4 SV=1 - [A0A0K0D3U9_ANGCA]	1,303E8	539,22	133	14,7	0,60%
A0A0K0CTF4	Glutathione peroxidase OS=*Angiostrongylus cantonensis* PE=3 SV=1 - [A0A0K0CTF4_ANGCA]	5,096E7	427,89	166	18,7	0,57%
A0A158P7E8	Uncharacterised protein OS=*Angiostrongylus cantonensis* PE=3 SV=1 - [A0A158P7E8_ANGCA]	3,426E7	868,13	835	95,0	0,57%
A0A0K0DMY2	Uncharacterised protein OS=*Angiostrongylus cantonensis* PE=4 SV=1 - [A0A0K0DMY2_ANGCA]	2,186E8	645,15	143	16,3	0,57%
A0A0K0DK72	Uncharacterised protein OS=*Angiostrongylus cantonensis* PE=3 SV=1 - [A0A0K0DK72_ANGCA]	4,093E7	520,37	138	15,7	0,55%
A0A0K0D931	Uncharacterised protein OS=*Angiostrongylus cantonensis* PE=4 SV=1 - [A0A0K0D931_ANGCA]	2,202E7	622,13	185	20,4	0,55%
A0A0K0D967	Uncharacterised protein OS=*Angiostrongylus cantonensis* PE=4 SV=1 - [A0A0K0D967_ANGCA]	9,064E8	734,39	163	18,5	0,55%
A0A0K0DCN2	Uncharacterised protein OS=*Angiostrongylus cantonensis* PE=4 SV=1 - [A0A0K0DCN2_ANGCA]	1,466E7	634,92	545	62,1	0,55%
A0A0K0CYD1	Uncharacterised protein OS=*Angiostrongylus cantonensis* PE=3 SV=1 - [A0A0K0CYD1_ANGCA]	3,264E7	551,22	660	73,2	0,50%
A0A0K0D2N6	Uncharacterised protein OS=*Angiostrongylus cantonensis* PE=4 SV=1 - [A0A0K0D2N6_ANGCA]	3,829E7	539,78	471	50,6	0,50%
A0A158P6G0	Uncharacterised protein OS=*Angiostrongylus cantonensis* PE=4 SV=1 - [A0A158P6G0_ANGCA]	2,253E7	394,26	446	50,2	0,50%
A0A158P7V7	Uncharacterised protein OS=*Angiostrongylus cantonensis* PE=4 SV=1 - [A0A158P7V7_ANGCA]	2,166E7	733,44	429	48,3	0,50%
A0A0K0DIQ5	Superoxide dismutase [Cu-Zn] OS=*Angiostrongylus cantonensis* PE=3 SV=1 - [A0A0K0DIQ5_ANGCA]	1,351E8	470,73	159	16,4	0,48%
A0A158P857	Uncharacterised protein OS=*Angiostrongylus cantonensis* PE=3 SV=1 - [A0A158P857_ANGCA]	7,660E6	609,76	802	89,8	0,48%

Accession	Description	Area	Score	# AAs	MW [kDa]	Relative protein abundance [%]
A0A0K0DA83	Uncharacterised protein OS=*Angiostrongylus cantonensis* PE=4 SV=1 - [A0A0K0DA83_ANGCA]	1,411E8	478,90	120	13,2	0,48%
A0A158PAM4	Uncharacterised protein OS=*Angiostrongylus cantonensis* PE=4 SV=1 - [A0A158PAM4_ANGCA]	2,857E7	462,77	303	34,9	0,45%
A0A0K0DK12	Glyceraldehyde-3-phosphate dehydrogenase OS=*Angiostrongylus cantonensis* PE=3 SV=1 - [A0A0K0DK12_ANGCA]	2,739E7	625,99	341	36,5	0,43%
A0A158P864	Nucleoside diphosphate kinase OS=*Angiostrongylus cantonensis* PE=3 SV=1 - [A0A158P864_ANGCA]	9,637E7	542,62	153	17,4	0,43%
A0A0K0CYE0	Uncharacterised protein OS=*Angiostrongylus cantonensis* PE=4 SV=1 - [A0A0K0CYE0_ANGCA]	3,878E7	431,01	169	18,8	0,43%
A0A0K0DPX8	Uncharacterised protein OS=*Angiostrongylus cantonensis* PE=4 SV=1 - [A0A0K0DPX8_ANGCA]	1,529E7	370,14	430	50,5	0,43%
A0A0K0CTC6	Elongation factor 1-alpha OS=*Angiostrongylus cantonensis* PE=3 SV=1 - [A0A0K0CTC6_ANGCA]	1,474E7	441,65	464	50,5	0,41%
A0A0K0CT12	Uncharacterised protein OS=*Angiostrongylus cantonensis* PE=4 SV=1 - [A0A0K0CT12_ANGCA]	1,932E7	390,65	167	18,7	0,41%
A0A0K0CXW0	Uncharacterised protein OS=*Angiostrongylus cantonensis* PE=4 SV=1 - [A0A0K0CXW0_ANGCA]	1,358E7	329,86	616	66,6	0,41%
A0A0K0DBT5	Profilin OS=*Angiostrongylus cantonensis* PE=3 SV=1 - [A0A0K0DBT5_ANGCA]	4,318E7	668,30	163	17,7	0,38%
A0A158PCV1	Uncharacterised protein OS=*Angiostrongylus cantonensis* PE=3 SV=1 - [A0A158PCV1_ANGCA]	1,725E7	609,41	535	59,5	0,38%
A0A0K0DEA9	Uncharacterised protein OS=*Angiostrongylus cantonensis* PE=4 SV=1 - [A0A0K0DEA9_ANGCA]	4,106E7	548,86	220	23,0	0,38%
C7TNT7	Putative Uncharacterised protein (Fragment) OS=*Angiostrongylus cantonensis* PE=2 SV=1 - [C7TNT7_ANGCA]	1,456E8	395,36	121	13,6	0,36%
A0A0K0DHN4	Transaldolase OS=*Angiostrongylus cantonensis* PE=3 SV=1 - [A0A0K0DHN4_ANGCA]	1,677E7	543,66	321	36,0	0,36%
A0A0K0D5B3	Uncharacterised protein OS=*Angiostrongylus cantonensis* PE=4 SV=1 - [A0A0K0D5B3_ANGCA]	9,614E8	550,02	239	26,9	0,36%
A0A0K0DD77	Uncharacterised protein OS=*Angiostrongylus cantonensis* PE=4 SV=1 - [A0A0K0DD77_ANGCA]	2,641E7	327,78	226	24,6	0,36%
A0A0K0D9T8	Uncharacterised protein OS=*Angiostrongylus cantonensis* PE=4 SV=2 - [A0A0K0D9T8_ANGCA]	6,338E6	345,28	1499	164,0	0,36%
A0A0K0CZ46	Malate dehydrogenase OS=*Angiostrongylus cantonensis* PE=3 SV=1 - [A0A0K0CZ46_ANGCA]	3,611E7	336,67	337	36,1	0,33%
A0A0K0D5E2	Uncharacterised protein OS=*Angiostrongylus cantonensis* PE=4 SV=1 - [A0A0K0D5E2_ANGCA]	8,135E7	306,45	132	14,8	0,33%
A0A0K0DGK5	Uncharacterised protein OS=*Angiostrongylus cantonensis* PE=4 SV=1 - [A0A0K0DGK5_ANGCA]	4,698E7	315,69	252	28,2	0,33%
A0A0K0DAJ5	Uncharacterised protein OS=*Angiostrongylus cantonensis* PE=4 SV=2 - [A0A0K0DAJ5_ANGCA]	4,291E7	539,70	750	78,4	0,33%
A0A0K0D2L7	Peptidyl-prolyl cis-trans isomerase OS=*Angiostrongylus cantonensis* PE=3 SV=1 - [A0A0K0D2L7_ANGCA]	1,629E8	409,21	171	18,5	0,31%
A0A0K0CTT0	Tubulin alpha chain OS=*Angiostrongylus cantonensis* PE=3 SV=1 - [A0A0K0CTT0_ANGCA]	1,450E7	318,87	451	50,5	0,31%
A0A0K0DDP7	Uncharacterised protein OS=*Angiostrongylus cantonensis* PE=3 SV=1 - [A0A0K0DDP7_ANGCA]	3,947E7	343,03	397	45,8	0,31%
A0A158PAS4	Uncharacterised protein OS=*Angiostrongylus cantonensis* PE=3 SV=1 - [A0A158PAS4_ANGCA]	1,802E7	377,52	299	33,6	0,31%
A0A0K0D819	Uncharacterised protein OS=*Angiostrongylus cantonensis* PE=4 SV=1 - [A0A0K0D819_ANGCA]	2,542E7	247,37	111	13,0	0,31%
A0A158PCE0	Uncharacterised protein OS=*Angiostrongylus cantonensis* PE=4 SV=1 - [A0A158PCE0_ANGCA]	4,648E7	245,09	194	22,0	0,31%
A0A0K0DI98	Glucose-6-phosphate isomerase OS=*Angiostrongylus cantonensis* PE=3 SV=2 - [A0A0K0DI98_ANGCA]	2,682E7	322,15	553	62,0	0,29%
A0A0K0D128	Uncharacterised protein OS=*Angiostrongylus cantonensis* PE=3 SV=1 - [A0A0K0D128_ANGCA]	4,719E7	296,26	110	12,5	0,29%
A0A0K0D232	Uncharacterised protein OS=*Angiostrongylus cantonensis* PE=3 SV=1 - [A0A0K0D232_ANGCA]	9,988E6	264,30	449	48,6	0,29%
A0A158P939	Uncharacterised protein OS=*Angiostrongylus cantonensis* PE=3 SV=1 - [A0A158P939_ANGCA]	1,753E7	140,96	294	33,3	0,29%
A0A158P988	Uncharacterised protein OS=*Angiostrongylus cantonensis* PE=3 SV=1 - [A0A158P988_ANGCA]	1,768E7	272,50	706	80,0	0,29%

Accession	Description	Area	Score	# AAs	MW [kDa]	Relative protein abundance [%]
A0A0K0CVM5	Uncharacterised protein OS=*Angiostrongylus cantonensis* PE=4 SV=1 - [A0A0K0CVM5_ANGCA]	1,325E7	228,48	266	30,0	0,29%
A0A0K0D884	Uncharacterised protein OS=*Angiostrongylus cantonensis* PE=4 SV=1 - [A0A0K0D884_ANGCA]	1,260E7	100,73	117	13,7	0,29%
A0A0K0DB86	Uncharacterised protein OS=*Angiostrongylus cantonensis* PE=4 SV=1 - [A0A0K0DB86_ANGCA]	1,292E7	320,25	902	96,7	0,29%
A0A0K0DAC4	Aspartate aminotransferase OS=*Angiostrongylus cantonensis* PE=4 SV=1 - [A0A0K0DAC4_ANGCA]	1,169E7	308,92	408	45,8	0,26%
A0A0K0CT44	Major sperm protein OS=*Angiostrongylus cantonensis* PE=4 SV=1 - [A0A0K0CT44_ANGCA]	1,764E7	356,63	126	14,3	0,26%
A0A0K0DHF4	Peptidyl-prolyl cis-trans isomerase OS=*Angiostrongylus cantonensis* PE=3 SV=1 - [A0A0K0DHF4_ANGCA]	3,233E7	306,43	163	17,5	0,26%
A0A0K0DDQ6	Tubulin beta chain OS=*Angiostrongylus cantonensis* PE=3 SV=1 - [A0A0K0DDQ6_ANGCA]	9,838E6	320,61	449	50,1	0,26%
A0A0K0CW15	Uncharacterised protein OS=*Angiostrongylus cantonensis* PE=4 SV=1 - [A0A0K0CW15_ANGCA]	1,974E7	245,55	194	22,4	0,26%
A0A0K0CXK8	Uncharacterised protein OS=*Angiostrongylus cantonensis* PE=4 SV=1 - [A0A0K0CXK8_ANGCA]	1,845E7	454,72	198	22,7	0,26%
A0A0K0D287	Uncharacterised protein OS=*Angiostrongylus cantonensis* PE=4 SV=1 - [A0A0K0D287_ANGCA]	5,818E7	253,49	108	11,8	0,26%
A0A0K0D4I4	Uncharacterised protein OS=*Angiostrongylus cantonensis* PE=4 SV=1 - [A0A0K0D4I4_ANGCA]	7,526E6	375,39	318	35,3	0,26%
A0A0K0DGY9	Uncharacterised protein OS=*Angiostrongylus cantonensis* PE=4 SV=1 - [A0A0K0DGY9_ANGCA]	1,593E7	285,23	617	63,9	0,26%
A0A0K0DI57	Uncharacterised protein OS=*Angiostrongylus cantonensis* PE=4 SV=1 - [A0A0K0DI57_ANGCA]	1,493E8	326,85	130	14,7	0,26%
A0A0K0DQ80	Uncharacterised protein OS=*Angiostrongylus cantonensis* PE=4 SV=1 - [A0A0K0DQ80_ANGCA]	1,910E7	185,29	667	73,6	0,26%
A0A0K0D2C0	Pyruvate kinase OS=*Angiostrongylus cantonensis* PE=3 SV=1 - [A0A0K0D2C0_ANGCA]	1,301E7	140,16	475	51,6	0,24%
A0A0K0D3J9	Uncharacterised protein OS=*Angiostrongylus cantonensis* PE=4 SV=1 - [A0A0K0D3J9_ANGCA]	6,851E7	390,29	213	24,2	0,24%
A0A0K0DEG3	Uncharacterised protein OS=*Angiostrongylus cantonensis* PE=4 SV=1 - [A0A0K0DEG3_ANGCA]	3,808E7	255,37	145	16,2	0,24%
A0A0K0DEG6	Uncharacterised protein OS=*Angiostrongylus cantonensis* PE=4 SV=1 - [A0A0K0DEG6_ANGCA]	2,846E8	255,34	277	30,4	0,24%
A0A0K0DM49	Uncharacterised protein OS=*Angiostrongylus cantonensis* PE=4 SV=1 - [A0A0K0DM49_ANGCA]	1,560E7	193,40	166	19,6	0,24%
A0A0K0DME1	Uncharacterised protein OS=*Angiostrongylus cantonensis* PE=4 SV=1 - [A0A0K0DME1_ANGCA]	1,609E7	297,57	215	24,0	0,24%
A0A158P6C5	Uncharacterised protein OS=*Angiostrongylus cantonensis* PE=4 SV=1 - [A0A158P6C5_ANGCA]	1,133E7	145,93	1226	138,4	0,24%
A0A158P995	Uncharacterised protein OS=*Angiostrongylus cantonensis* PE=4 SV=1 - [A0A158P995_ANGCA]	9,648E6	258,46	2113	226,6	0,24%
A0A158PB07	Uncharacterised protein OS=*Angiostrongylus cantonensis* PE=4 SV=1 - [A0A158PB07_ANGCA]	1,202E7	246,02	426	47,2	0,24%
G1EUS8	NAC domain containing protein OS=*Angiostrongylus cantonensis* PE=2 SV=1 - [G1EUS8_ANGCA]	1,354E7	367,52	185	20,4	0,21%
A0A0K0DQR1	Peptidyl-prolyl cis-trans isomerase OS=*Angiostrongylus cantonensis* PE=3 SV=1 - [A0A0K0DQR1_ANGCA]	5,737E7	195,51	159	17,2	0,21%
A0A158P605	Uncharacterised protein OS=*Angiostrongylus cantonensis* PE=3 SV=1 - [A0A158P605_ANGCA]	1,460E7	254,42	226	25,8	0,21%
A0A0K0CYX0	Uncharacterised protein OS=*Angiostrongylus cantonensis* PE=4 SV=1 - [A0A0K0CYX0_ANGCA]	8,381E6	325,34	245	28,4	0,21%
A0A158P600	Uncharacterised protein OS=*Angiostrongylus cantonensis* PE=4 SV=1 - [A0A158P600_ANGCA]	9,264E6	222,13	528	59,3	0,21%
A0A158P7Y8	Uncharacterised protein OS=*Angiostrongylus cantonensis* PE=4 SV=1 - [A0A158P7Y8_ANGCA]	3,547E7	287,02	266	29,9	0,21%
A0A158PB85	Uncharacterised protein OS=*Angiostrongylus cantonensis* PE=4 SV=1 - [A0A158PB85_ANGCA]	5,713E6	113,86	200	22,7	0,21%
A0A0K0DKI8	Isocitrate dehydrogenase [NADP] OS=*Angiostrongylus cantonensis* PE=3 SV=1 - [A0A0K0DKI8_ANGCA]	6,180E6	282,60	395	44,4	0,19%
A0A0K0D2A0	Peptidyl-prolyl cis-trans isomerase OS=*Angiostrongylus cantonensis* PE=3 SV=1 - [A0A0K0D2A0_ANGCA]	1,212E8	234,44	178	19,6	0,19%
A0A0K0CW08	Uncharacterised protein OS=*Angiostrongylus cantonensis* PE=4 SV=1 - [A0A0K0CW08_ANGCA]	7,340E6	181,35	264	29,8	0,19%

Accession	Description	Area	Score	# AAs	MW [kDa]	Relative protein abundance [%]
A0A0K0D904	Uncharacterised protein OS=*Angiostrongylus cantonensis* PE=4 SV=1 - [A0A0K0D904_ANGCA]	4,652E6	195,79	438	48,9	0,19%
A0A158P622	Uncharacterised protein OS=*Angiostrongylus cantonensis* PE=4 SV=1 - [A0A158P622_ANGCA]	1,086E7	238,41	567	64,6	0,19%
A0A158PCK6	Uncharacterised protein OS=*Angiostrongylus cantonensis* PE=4 SV=1 - [A0A158PCK6_ANGCA]	8,044E6	160,27	198	22,3	0,19%
A0A0K0D311	Eukaryotic translation initiation factor 5A OS=*Angiostrongylus cantonensis* PE=3 SV=1 - [A0A0K0D311_ANGCA]	4,982E7	220,58	145	16,0	0,17%
A0A0K0DAB9	Uncharacterised protein OS=*Angiostrongylus cantonensis* PE=3 SV=1 - [A0A0K0DAB9_ANGCA]	4,639E6	214,71	394	44,5	0,17%
A0A158P8Y6	Uncharacterised protein OS=*Angiostrongylus cantonensis* PE=3 SV=1 - [A0A158P8Y6_ANGCA]	2,578E7	85,14	292	33,3	0,17%
A0A158P9S5	Uncharacterised protein OS=*Angiostrongylus cantonensis* PE=3 SV=1 - [A0A158P9S5_ANGCA]	9,892E6	317,43	363	40,8	0,17%
A0A158PAS3	Uncharacterised protein OS=*Angiostrongylus cantonensis* PE=3 SV=1 - [A0A158PAS3_ANGCA]	8,425E6	285,17	356	39,9	0,17%
A0A0K0D9G9	Uncharacterised protein OS=*Angiostrongylus cantonensis* PE=3 SV=2 - [A0A0K0D9G9_ANGCA]	1,373E7	160,07	114	12,3	0,17%
A0A0K0CXT9	Uncharacterised protein OS=*Angiostrongylus cantonensis* PE=4 SV=1 - [A0A0K0CXT9_ANGCA]	7,813E6	217,25	135	15,3	0,17%
A0A0K0DB82	Uncharacterised protein OS=*Angiostrongylus cantonensis* PE=4 SV=1 - [A0A0K0DB82_ANGCA]	8,179E6	191,70	459	50,0	0,17%
A0A0K0DGH7	Uncharacterised protein OS=*Angiostrongylus cantonensis* PE=4 SV=1 - [A0A0K0DGH7_ANGCA]	4,044E6	185,47	385	44,2	0,17%
A0A0K0DMB8	Uncharacterised protein OS=*Angiostrongylus cantonensis* PE=4 SV=1 - [A0A0K0DMB8_ANGCA]	2,260E8	149,61	153	16,3	0,17%
A0A0K0DN78	Uncharacterised protein OS=*Angiostrongylus cantonensis* PE=4 SV=1 - [A0A0K0DN78_ANGCA]	2,508E7	296,76	117	13,2	0,17%
A0A0K0DQM7	Uncharacterised protein OS=*Angiostrongylus cantonensis* PE=4 SV=1 - [A0A0K0DQM7_ANGCA]	1,036E7	55,29	336	40,2	0,17%
A0A158P6V6	Uncharacterised protein OS=*Angiostrongylus cantonensis* PE=4 SV=1 - [A0A158P6V6_ANGCA]	1,316E7	145,65	720	82,7	0,17%
A0A158PB54	Uncharacterised protein OS=*Angiostrongylus cantonensis* PE=4 SV=1 - [A0A158PB54_ANGCA]	1,430E7	160,02	509	56,8	0,17%
A0A158PBD9	Uncharacterised protein OS=*Angiostrongylus cantonensis* PE=4 SV=1 - [A0A158PBD9_ANGCA]	8,021E6	51,09	608	69,2	0,17%
A0A158P8B8	L-lactate dehydrogenase OS=*Angiostrongylus cantonensis* PE=3 SV=1 - [A0A158P8B8_ANGCA]	1,644E7	202,20	338	36,6	0,14%
A0A0K0CY27	Uncharacterised protein OS=*Angiostrongylus cantonensis* PE=3 SV=1 - [A0A0K0CY27_ANGCA]	6,519E6	174,16	181	19,8	0,14%
A0A0K0D2E9	Uncharacterised protein OS=*Angiostrongylus cantonensis* PE=3 SV=1 - [A0A0K0D2E9_ANGCA]	1,203E7	200,29	216	24,4	0,14%
A0A0K0CVY2	Uncharacterised protein OS=*Angiostrongylus cantonensis* PE=4 SV=1 - [A0A0K0CVY2_ANGCA]	1,573E7	228,53	156	17,4	0,14%
A0A0K0CWR0	Uncharacterised protein OS=*Angiostrongylus cantonensis* PE=4 SV=1 - [A0A0K0CWR0_ANGCA]	3,473E7	214,35	117	13,2	0,14%
A0A0K0CZE8	Uncharacterised protein OS=*Angiostrongylus cantonensis* PE=4 SV=1 - [A0A0K0CZE8_ANGCA]	1,892E7	168,88	166	19,3	0,14%
A0A0K0CZQ0	Uncharacterised protein OS=*Angiostrongylus cantonensis* PE=4 SV=1 - [A0A0K0CZQ0_ANGCA]	6,623E6	190,28	301	34,5	0,14%
A0A0K0D2H9	Uncharacterised protein OS=*Angiostrongylus cantonensis* PE=4 SV=1 - [A0A0K0D2H9_ANGCA]	1,159E7	129,22	404	46,3	0,14%
A0A0K0D8Z8	Uncharacterised protein OS=*Angiostrongylus cantonensis* PE=4 SV=1 - [A0A0K0D8Z8_ANGCA]	1,048E7	236,01	127	14,8	0,14%
A0A0K0DC10	Uncharacterised protein OS=*Angiostrongylus cantonensis* PE=4 SV=1 - [A0A0K0DC10_ANGCA]	5,116E6	194,31	163	18,4	0,14%
A0A0K0DFP3	Uncharacterised protein OS=*Angiostrongylus cantonensis* PE=4 SV=1 - [A0A0K0DFP3_ANGCA]	1,310E7	218,72	207	23,9	0,14%
A0A0K0DHU3	Uncharacterised protein OS=*Angiostrongylus cantonensis* PE=4 SV=1 - [A0A0K0DHU3_ANGCA]	1,421E7	274,31	259	28,0	0,14%
A0A0K0DIJ9	Uncharacterised protein OS=*Angiostrongylus cantonensis* PE=4 SV=1 - [A0A0K0DIJ9_ANGCA]	8,550E6	118,23	185	21,1	0,14%
A0A0K0DIY2	Uncharacterised protein OS=*Angiostrongylus cantonensis* PE=4 SV=1 - [A0A0K0DIY2_ANGCA]	1,508E7	167,69	159	18,6	0,14%

Accession	Description	Area	Score	# AAs	MW [kDa]	Relative protein abundance [%]
A0A0K0DKI5	Uncharacterised protein OS=*Angiostrongylus cantonensis* PE=4 SV=1 - [A0A0K0DKI5_ANGCA]	1,308E7	76,82	220	24,3	0,14%
A0A0K0DLP8	Uncharacterised protein OS=*Angiostrongylus cantonensis* PE=4 SV=1 - [A0A0K0DLP8_ANGCA]	1,412E7	78,81	150	18,1	0,14%
A0A0K0DM98	Uncharacterised protein OS=*Angiostrongylus cantonensis* PE=4 SV=1 - [A0A0K0DM98_ANGCA]	1,950E7	102,52	110	12,4	0,14%
A0A0K0DNM4	Uncharacterised protein OS=*Angiostrongylus cantonensis* PE=4 SV=1 - [A0A0K0DNM4_ANGCA]	1,151E7	191,15	171	19,1	0,14%
A0A158P7G1	Uncharacterised protein OS=*Angiostrongylus cantonensis* PE=4 SV=1 - [A0A158P7G1_ANGCA]	1,251E7	219,84	141	15,2	0,14%
A0A158P8M9	Uncharacterised protein OS=*Angiostrongylus cantonensis* PE=4 SV=1 - [A0A158P8M9_ANGCA]	5,707E6	149,34	1664	191,1	0,14%
A0A158P9T3	Uncharacterised protein OS=*Angiostrongylus cantonensis* PE=4 SV=1 - [A0A158P9T3_ANGCA]	8,242E6	47,84	587	68,9	0,14%
A0A158PAY7	Uncharacterised protein OS=*Angiostrongylus cantonensis* PE=4 SV=1 - [A0A158PAY7_ANGCA]	8,660E6	213,41	162	18,1	0,14%
R4UYY8	Putative aspartic protease OS=*Angiostrongylus cantonensis* PE=2 SV=1 - [R4UYY8_ANGCA]	1,719E7	330,71	425	46,2	0,12%
C7BVR2	Putative Thyroglobulin type-1 repeat family protein OS=*Angiostrongylus cantonensis* GN=00012G02 PE=2 SV=1 - [C7BVR2_ANGCA]	6,780E6	95,52	285	33,0	0,12%
A0A0K0CXC1	Uncharacterised protein OS=*Angiostrongylus cantonensis* PE=3 SV=1 - [A0A0K0CXC1_ANGCA]	6,320E6	85,88	791	88,2	0,12%
A0A158P9A1	Uncharacterised protein OS=*Angiostrongylus cantonensis* PE=3 SV=1 - [A0A158P9A1_ANGCA]	7,269E6	86,43	256	29,6	0,12%
A0A158PB99	Uncharacterised protein OS=*Angiostrongylus cantonensis* PE=3 SV=1 - [A0A158PB99_ANGCA]	6,368E6	44,43	395	44,5	0,12%
A0A0K0CYF0	Uncharacterised protein OS=*Angiostrongylus cantonensis* PE=4 SV=1 - [A0A0K0CYF0_ANGCA]	9,214E6	102,90	404	45,4	0,12%
A0A0K0CZE1	Uncharacterised protein OS=*Angiostrongylus cantonensis* PE=4 SV=1 - [A0A0K0CZE1_ANGCA]	1,152E7	112,92	1605	186,9	0,12%
A0A0K0D079	Uncharacterised protein OS=*Angiostrongylus cantonensis* PE=4 SV=1 - [A0A0K0D079_ANGCA]	5,469E6	160,61	161	18,8	0,12%
A0A0K0D2L5	Uncharacterised protein OS=*Angiostrongylus cantonensis* PE=4 SV=1 - [A0A0K0D2L5_ANGCA]	3,506E6	143,16	309	35,4	0,12%
A0A0K0D4B5	Uncharacterised protein OS=*Angiostrongylus cantonensis* PE=4 SV=1 - [A0A0K0D4B5_ANGCA]	1,870E7	134,30	127	14,1	0,12%
A0A0K0D5T6	Uncharacterised protein OS=*Angiostrongylus cantonensis* PE=4 SV=1 - [A0A0K0D5T6_ANGCA]	1,731E7	147,97	278	30,3	0,12%
A0A0K0D6W5	Uncharacterised protein OS=*Angiostrongylus cantonensis* PE=4 SV=1 - [A0A0K0D6W5_ANGCA]	2,033E7	169,10	68	7,7	0,12%
A0A0K0D835	Uncharacterised protein OS=*Angiostrongylus cantonensis* PE=4 SV=1 - [A0A0K0D835_ANGCA]	4,332E7	186,78	101	11,2	0,12%
A0A0K0DB40	Uncharacterised protein OS=*Angiostrongylus cantonensis* PE=4 SV=1 - [A0A0K0DB40_ANGCA]	8,166E6	68,65	471	52,7	0,12%
A0A0K0DG68	Uncharacterised protein OS=*Angiostrongylus cantonensis* PE=4 SV=1 - [A0A0K0DG68_ANGCA]	6,842E6	162,90	253	28,2	0,12%
A0A0K0DIE8	Uncharacterised protein OS=*Angiostrongylus cantonensis* PE=4 SV=1 - [A0A0K0DIE8_ANGCA]	5,552E6	119,78	601	69,4	0,12%
A0A0K0DRB5	Uncharacterised protein OS=*Angiostrongylus cantonensis* PE=4 SV=1 - [A0A0K0DRB5_ANGCA]	1,499E6	42,21	168	18,4	0,12%
A0A158P7W7	Uncharacterised protein OS=*Angiostrongylus cantonensis* PE=4 SV=1 - [A0A158P7W7_ANGCA]	7,049E6	155,85	289	31,7	0,12%
A0A158P8U4	Uncharacterised protein OS=*Angiostrongylus cantonensis* PE=4 SV=1 - [A0A158P8U4_ANGCA]	2,913E6	99,66	448	49,5	0,12%
A0A158P9E2	Uncharacterised protein OS=*Angiostrongylus cantonensis* PE=4 SV=1 - [A0A158P9E2_ANGCA]	3,672E6	60,25	159	17,8	0,12%
A0A158P9L0	Uncharacterised protein OS=*Angiostrongylus cantonensis* PE=4 SV=1 - [A0A158P9L0_ANGCA]	1,288E7	166,53	326	35,5	0,12%
A0A0K0D1Y8	Carboxypeptidase OS=*Angiostrongylus cantonensis* PE=3 SV=1 - [A0A0K0D1Y8_ANGCA]	8,820E6	81,47	342	38,9	0,10%
A0A0K0D9R8	L-lactate dehydrogenase OS=*Angiostrongylus cantonensis* PE=3 SV=1 - [A0A0K0D9R8_ANGCA]	7,580E6	138,01	328	35,9	0,10%
C7BVV9	Putative CU (Copper) Chaperonin OS=*Angiostrongylus cantonensis* PE=2 SV=1 - [C7BVV9_ANGCA]	7,172E6	150,87	69	7,6	0,10%

Accession	Description	Area	Score	# AAs	MW [kDa]	Relative protein abundance [%]
A0A0K0CZL3	Uncharacterised protein OS=*Angiostrongylus cantonensis* PE=3 SV=1 - [A0A0K0CZL3_ANGCA]	4,725E6	82,86	531	57,5	0,10%
A0A0K0DFZ4	Uncharacterised protein OS=*Angiostrongylus cantonensis* PE=3 SV=1 - [A0A0K0DFZ4_ANGCA]	1,433E7	273,87	88	9,5	0,10%
A0A0K0DJC2	Uncharacterised protein OS=*Angiostrongylus cantonensis* PE=3 SV=1 - [A0A0K0DJC2_ANGCA]	5,708E6	90,48	151	17,7	0,10%
A0A0K0CXF8	Uncharacterised protein OS=*Angiostrongylus cantonensis* PE=4 SV=1 - [A0A0K0CXF8_ANGCA]	6,912E6	108,72	276	31,0	0,10%
A0A0K0D4D5	Uncharacterised protein OS=*Angiostrongylus cantonensis* PE=4 SV=1 - [A0A0K0D4D5_ANGCA]	9,013E6	129,00	284	31,9	0,10%
A0A0K0DA48	Uncharacterised protein OS=*Angiostrongylus cantonensis* PE=4 SV=1 - [A0A0K0DA48_ANGCA]	5,454E6	22,32	367	40,8	0,10%
A0A0K0DBY6	Uncharacterised protein OS=*Angiostrongylus cantonensis* PE=4 SV=1 - [A0A0K0DBY6_ANGCA]	3,456E6	82,71	193	22,4	0,10%
A0A0K0DC77	Uncharacterised protein OS=*Angiostrongylus cantonensis* PE=4 SV=1 - [A0A0K0DC77_ANGCA]	4,129E7	98,48	76	8,5	0,10%
A0A0K0DEM7	Uncharacterised protein OS=*Angiostrongylus cantonensis* PE=4 SV=1 - [A0A0K0DEM7_ANGCA]	5,890E6	202,48	332	38,4	0,10%
A0A0K0DFN3	Uncharacterised protein OS=*Angiostrongylus cantonensis* PE=4 SV=1 - [A0A0K0DFN3_ANGCA]	5,307E6	200,15	105	11,3	0,10%
A0A0K0DJ36	Uncharacterised protein OS=*Angiostrongylus cantonensis* PE=4 SV=1 - [A0A0K0DJ36_ANGCA]	9,344E6	93,47	246	28,3	0,10%
A0A0K0DJ53	Uncharacterised protein OS=*Angiostrongylus cantonensis* PE=4 SV=1 - [A0A0K0DJ53_ANGCA]	1,527E7	120,40	231	25,3	0,10%
A0A0K0DMX9	Uncharacterised protein OS=*Angiostrongylus cantonensis* PE=4 SV=1 - [A0A0K0DMX9_ANGCA]	5,198E6	92,51	180	21,0	0,10%
A0A0K0DRN1	Uncharacterised protein OS=*Angiostrongylus cantonensis* PE=4 SV=1 - [A0A0K0DRN1_ANGCA]	4,995E6	84,31	447	50,6	0,10%
A0A158P8C2	Uncharacterised protein OS=*Angiostrongylus cantonensis* PE=4 SV=1 - [A0A158P8C2_ANGCA]	6,120E6	113,66	326	36,6	0,10%
A0A158P8K3	Uncharacterised protein OS=*Angiostrongylus cantonensis* PE=4 SV=1 - [A0A158P8K3_ANGCA]	4,066E6	72,27	407	44,4	0,10%
A0A158P979	Uncharacterised protein OS=*Angiostrongylus cantonensis* PE=4 SV=1 - [A0A158P979_ANGCA]	9,628E6	193,01	126	14,7	0,10%
A0A0K0DGT4	Uncharacterised protein OS=*Angiostrongylus cantonensis* PE=4 SV=3 - [A0A0K0DGT4_ANGCA]	9,548E6	122,24	385	42,8	0,10%
A0A158PAL5	Aspartate aminotransferase OS=*Angiostrongylus cantonensis* PE=4 SV=1 - [A0A158PAL5_ANGCA]	3,425E6	48,86	392	43,2	0,07%
G1EUS1	Galectin OS=*Angiostrongylus cantonensis* PE=2 SV=1 - [G1EUS1_ANGCA]	8,987E6	45,79	285	32,6	0,07%
C7BVQ3	Putative Tubulin-specific chaperone A OS=*Angiostrongylus cantonensis* GN=cfa PE=2 SV=1 - [C7BVQ3_ANGCA]	8,041E6	56,15	112	12,8	0,07%
C7BVL3	Putative Uncharacterised protein OS=*Angiostrongylus cantonensis* PE=2 SV=1 - [C7BVL3_ANGCA]	3,161E7	118,53	64	7,0	0,07%
A0A0K0DET6	Small ubiquitin-related modifier OS=*Angiostrongylus cantonensis* PE=3 SV=1 - [A0A0K0DET6_ANGCA]	3,378E6	89,64	96	10,5	0,07%
A0A158P6N9	Ubiquitin carboxyl-terminal hydrolase OS=*Angiostrongylus cantonensis* PE=3 SV=1 - [A0A158P6N9_ANGCA]	3,680E6	96,61	435	47,5	0,07%
A0A0K0D0G7	Uncharacterised protein OS=*Angiostrongylus cantonensis* PE=3 SV=1 - [A0A0K0D0G7_ANGCA]	5,569E6	44,77	147	17,3	0,07%
A0A0K0CT30	Uncharacterised protein OS=*Angiostrongylus cantonensis* PE=4 SV=1 - [A0A0K0CT30_ANGCA]	8,263E6	65,40	139	15,7	0,07%
A0A0K0CVM0	Uncharacterised protein OS=*Angiostrongylus cantonensis* PE=4 SV=1 - [A0A0K0CVM0_ANGCA]	3,278E6	102,61	460	51,4	0,07%
A0A0K0CWS0	Uncharacterised protein OS=*Angiostrongylus cantonensis* PE=4 SV=1 - [A0A0K0CWS0_ANGCA]	7,554E6	33,84	266	31,3	0,07%
A0A0K0CXF6	Uncharacterised protein OS=*Angiostrongylus cantonensis* PE=4 SV=1 - [A0A0K0CXF6_ANGCA]	2,931E6	175,46	166	18,0	0,07%
A0A0K0D0Y4	Uncharacterised protein OS=*Angiostrongylus cantonensis* PE=4 SV=1 - [A0A0K0D0Y4_ANGCA]	6,124E6	119,45	228	25,1	0,07%
A0A0K0D1C9	Uncharacterised protein OS=*Angiostrongylus cantonensis* PE=4 SV=1 - [A0A0K0D1C9_ANGCA]	5,010E6	60,12	220	23,6	0,07%
A0A0K0DCZ0	Uncharacterised protein OS=*Angiostrongylus cantonensis* PE=4 SV=1 - [A0A0K0DCZ0_ANGCA]	6,033E6	34,79	152	17,2	0,07%

Accession	Description	Area	Score	# AAs	MW [kDa]	Relative protein abundance [%]
A0A0K0DD39	Uncharacterised protein OS=*Angiostrongylus cantonensis* PE=4 SV=1 - [A0A0K0DD39_ANGCA]	4,489E6	75,22	198	21,7	0,07%
A0A0K0DE51	Uncharacterised protein OS=*Angiostrongylus cantonensis* PE=4 SV=1 - [A0A0K0DE51_ANGCA]	9,494E6	123,27	196	20,9	0,07%
A0A0K0DEG2	Uncharacterised protein OS=*Angiostrongylus cantonensis* PE=4 SV=1 - [A0A0K0DEG2_ANGCA]	1,090E7	85,69	145	15,7	0,07%
A0A0K0DEX3	Uncharacterised protein OS=*Angiostrongylus cantonensis* PE=4 SV=1 - [A0A0K0DEX3_ANGCA]	6,378E6	33,00	129	14,4	0,07%
A0A0K0DG91	Uncharacterised protein OS=*Angiostrongylus cantonensis* PE=4 SV=1 - [A0A0K0DG91_ANGCA]	6,374E6	101,99	299	30,1	0,07%
A0A0K0DGJ9	Uncharacterised protein OS=*Angiostrongylus cantonensis* PE=4 SV=1 - [A0A0K0DGJ9_ANGCA]	6,766E6	38,45	625	70,5	0,07%
A0A0K0DJD3	Uncharacterised protein OS=*Angiostrongylus cantonensis* PE=4 SV=1 - [A0A0K0DJD3_ANGCA]	9,609E6	122,71	54	6,1	0,07%
A0A158P8L2	Uncharacterised protein OS=*Angiostrongylus cantonensis* PE=4 SV=1 - [A0A158P8L2_ANGCA]	2,810E6	109,33	152	17,5	0,07%
A0A158PCB0	Uncharacterised protein OS=*Angiostrongylus cantonensis* PE=4 SV=1 - [A0A158PCB0_ANGCA]	6,328E6	99,55	149	17,3	0,07%
A0A0K0CUQ2	40S ribosomal protein S12 OS=*Angiostrongylus cantonensis* PE=3 SV=1 - [A0A0K0CUQ2_ANGCA]	3,112E6	63,85	139	15,1	0,05%
A0A158PBI7	Galectin OS=*Angiostrongylus cantonensis* PE=4 SV=1 - [A0A158PBI7_ANGCA]	3,950E6	57,12	278	31,6	0,05%
A0A158PBR8	Profilin OS=*Angiostrongylus cantonensis* PE=3 SV=1 - [A0A158PBR8_ANGCA]	4,434E6	51,07	126	13,4	0,05%
A0A0K0DD64	Proteasome subunit alpha type OS=*Angiostrongylus cantonensis* PE=3 SV=1 - [A0A0K0DD64_ANGCA]	2,633E6	33,35	250	27,8	0,05%
A0A0K0D4T6	Superoxide dismutase [Cu-Zn] OS=*Angiostrongylus cantonensis* PE=3 SV=1 - [A0A0K0D4T6_ANGCA]	6,189E6	78,19	180	19,3	0,05%
A0A0K0DNF0	Uncharacterised protein OS=*Angiostrongylus cantonensis* PE=3 SV=1 - [A0A0K0DNF0_ANGCA]	2,560E6	42,47	533	59,6	0,05%
A0A0K0CSR9	Uncharacterised protein OS=*Angiostrongylus cantonensis* PE=4 SV=1 - [A0A0K0CSR9_ANGCA]	7,850E6	74,97	88	9,8	0,05%
A0A0K0CTC8	Uncharacterised protein OS=*Angiostrongylus cantonensis* PE=4 SV=1 - [A0A0K0CTC8_ANGCA]	4,445E6	46,58	226	25,3	0,05%
A0A0K0CW78	Uncharacterised protein OS=*Angiostrongylus cantonensis* PE=4 SV=1 - [A0A0K0CW78_ANGCA]	3,927E6	47,10	411	44,7	0,05%
A0A0K0CY07	Uncharacterised protein OS=*Angiostrongylus cantonensis* PE=4 SV=1 - [A0A0K0CY07_ANGCA]	1,095E6	57,67	147	16,7	0,05%
A0A0K0D0Q2	Uncharacterised protein OS=*Angiostrongylus cantonensis* PE=4 SV=1 - [A0A0K0D0Q2_ANGCA]	1,544E6	59,84	214	23,6	0,05%
A0A0K0D1S7	Uncharacterised protein OS=*Angiostrongylus cantonensis* PE=4 SV=1 - [A0A0K0D1S7_ANGCA]	4,162E6	32,41	155	17,4	0,05%
A0A0K0D2Z3	Uncharacterised protein OS=*Angiostrongylus cantonensis* PE=4 SV=1 - [A0A0K0D2Z3_ANGCA]	9,692E6	52,04	89	10,4	0,05%
A0A0K0D8Y9	Uncharacterised protein OS=*Angiostrongylus cantonensis* PE=4 SV=1 - [A0A0K0D8Y9_ANGCA]	2,223E6	29,12	153	16,6	0,05%
A0A0K0DBC0	Uncharacterised protein OS=*Angiostrongylus cantonensis* PE=4 SV=1 - [A0A0K0DBC0_ANGCA]	2,839E6	22,65	133	15,0	0,05%
A0A0K0DHB8	Uncharacterised protein OS=*Angiostrongylus cantonensis* PE=4 SV=1 - [A0A0K0DHB8_ANGCA]	6,715E5	79,34	113	11,9	0,05%
A0A0K0DNY1	Uncharacterised protein OS=*Angiostrongylus cantonensis* PE=4 SV=1 - [A0A0K0DNY1_ANGCA]	3,811E6	36,36	261	33,2	0,05%
A0A158P5R7	Uncharacterised protein OS=*Angiostrongylus cantonensis* PE=4 SV=1 - [A0A158P5R7_ANGCA]	5,558E6	28,97	416	45,8	0,05%
A0A158P9U6	Uncharacterised protein OS=*Angiostrongylus cantonensis* PE=4 SV=1 - [A0A158P9U6_ANGCA]	1,598E6	40,71	783	87,1	0,05%
A0A158PA25	Uncharacterised protein OS=*Angiostrongylus cantonensis* PE=4 SV=1 - [A0A158PA25_ANGCA]	2,017E6	87,79	805	89,2	0,05%
A0A158PBT8	Uncharacterised protein OS=*Angiostrongylus cantonensis* PE=4 SV=1 - [A0A158PBT8_ANGCA]	5,153E6	34,16	154	17,3	0,05%
A0A0K0DBG3	Uncharacterized protein OS=*Angiostrongylus cantonensis* PE=4 SV=2 - [A0A0K0DBG3_ANGCA]	5,561E6	79,43	885	100,3	0,05%

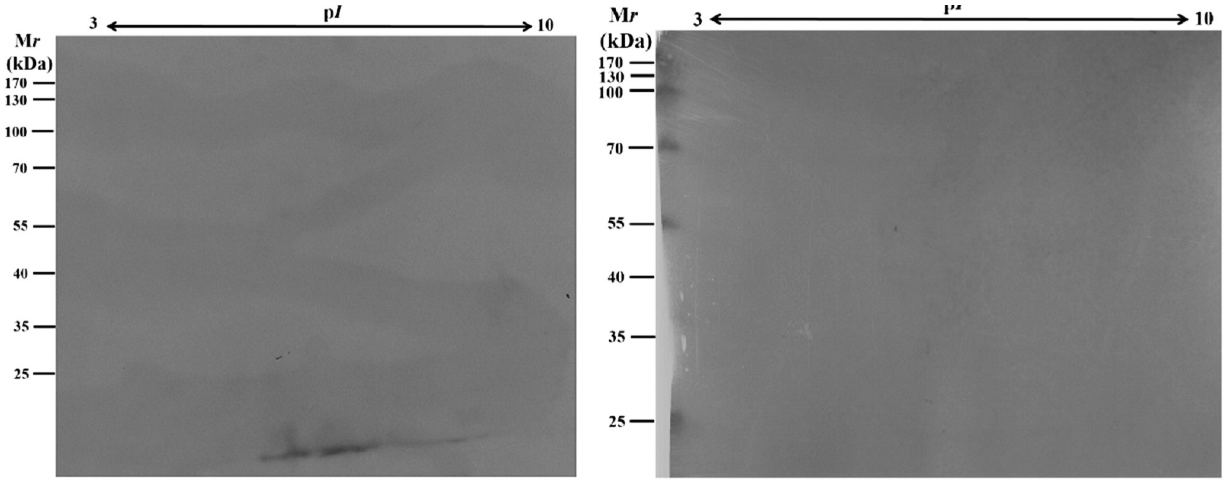

No mouse serum

Uninfected mouse serum

Western blotting analysis of the excretory-secretory products (ESPs) of *Angiostrongylus cantonensis* young adults against uninfected mouse serum or under mouse serum-free conditions.
